# A novel polymer enabled by polymerized small molecule strategy for tumor photothermal and photodynamic therapy

**DOI:** 10.1186/s12951-023-02272-9

**Published:** 2023-12-20

**Authors:** Xin Xie, Ke Wang, Jie Zeng, Miao-Yan Xu, Xin-Hui Qu, Zheng-Bin Xiang, Fang-Fang Tou, Shaorong Huang, Xiao-Jian Han

**Affiliations:** 1 School of Pharmacy, Jiangxi Medical College, Nanchang University, Nanchang, 330006 Jiangxi People’s Republic of China; 2 Institute of Geriatrics, Jiangxi Provincial People’s Hospital &, The First Affiliated Hospital of Nanchang Medical College, Nanchang, 330006 Jiangxi People’s Republic of China; 3 Department of Clinical Laboratory, Jiangxi Provincial Children’s Hospital, Nanchang, 330006 Jiangxi People’s Republic of China; 4 The Second Department of Neurology, Jiangxi Provincial People’s Hospital, The First Affiliated Hospital of Nanchang Medical College, Nanchang, 330006 Jiangxi People’s Republic of China; 5 Department of Oncology, Jiangxi Provincial People’s Hospital, The First Affiliated Hospital of Nanchang Medical College, Nanchang, 330006 Jiangxi People’s Republic of China

**Keywords:** Fused-ring molecule, Polymerized small molecule, Photothermal therapy, Photodynamic therapy, Tumor treatment

## Abstract

Photothermal therapy (PTT) and photodynamic therapy (PDT) are effective method for tumor treatment. However, the limited variety and quantity of photothermal agents (PTAs) and photosensitizer (PSs) are still major challenges. Moreover, the cell apoptosis mechanism induced by PDT and PTT is still elusive. A fused-ring small molecule acceptor–donor acceptor′ donor–acceptor (A-DA′D-A) type of Y5 (Scheme 1) has a narrow band-gap and strong light absorption. Herein, we used Y5 to polymerize with thiophene unit to obtain polymer PYT based on polymerized small molecule strategy, and PYT nanoparticles (PYT NPs) was prepared via one-step nanoprecipitation strategy with DSPE-PEG_2000_. PYT NPs had excellent biocompatibility, good photostability, high photothermal conversion efficiency (67%) and reactive oxygen species (ROS) production capacity under 808 nm laser irradiation (PYT NPs + NIR). In vitro and in vivo experiments revealed that PYT NPs + NIR had the ability to completely ablate tumor cells. It was demonstrated that cell apoptosis induced by PYT NPs + NIR was closely related to mitochondrial damage. This study provides valuable guidance for constructing high-performance organic PTAs and PSs for tumor treatment.

**Supplementary Information:**

The online version contains supplementary material available at 10.1186/s12951-023-02272-9.

## Highlights

1. Y5 molecule was polymerized with thiophene unit to synthesize PYT by polymerized small molecule strategy.
2. PYT nanoparticles (PYT NPs) was prepared via one-step nanoprecipitation strategy with DSPE-PEG_2000_.
3. PYT NPs had excellent performance of photothermal therapy, photodynamic therapy and fluorescence imaging for tumor.
4. Polymerized small molecule strategy is feasible to increase the variety and quantity of PTAs and PSs for the design of anti-tumor drugs.

**Supplementary Information:**

The online version contains supplementary material available at 10.1186/s12951-023-02272-9.

**Scheme 1:**
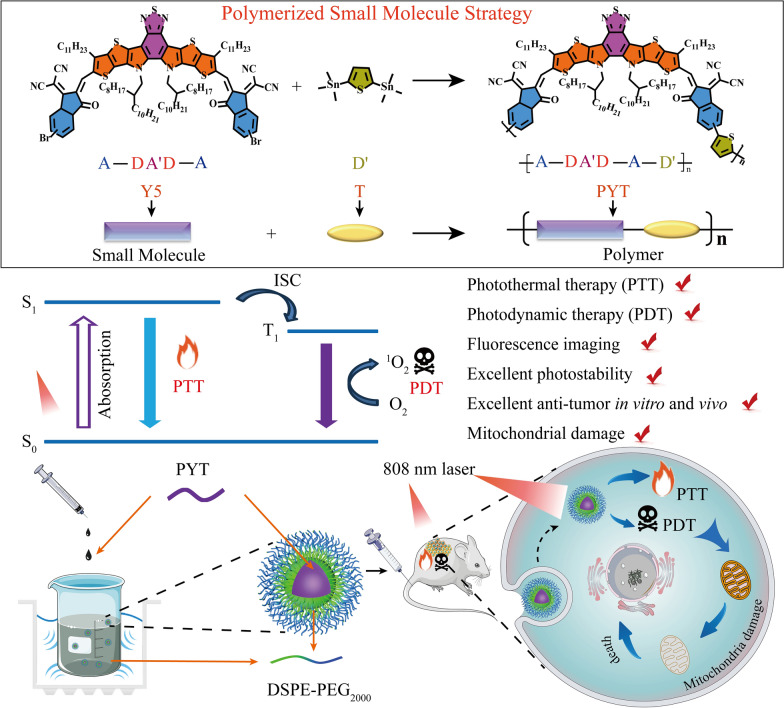
PYT enabled by polymerized small molecule strategy for tumor photothermal and photodynamic therapy

## Introduction

Cancer is one of the three major malignant diseases in the world [1, 2]. Radiotherapy, chemotherapy, and surgery are the traditional methods of treating tumors, which often have obvious toxic side effects on patients [3–5]. With the rapid development of materials science, photothermal therapy (PTT) and photodynamic therapy (PDT) have received widespread attention [6–8]. PTT is a therapeutic method to kill tumors by using the photothermal effect of photothermal agents (PTAs) [9, 10]. PDT is a therapeutic method that uses photosensitizer (PSs) and light irradiation to transform intracellular oxygen (O_2_) into toxic reactive oxygen species (ROS) to destroy tumor cells [11]. Since PTT and PDT possess low toxicity and non-invasiveness, which are potential tumor treatment methods. More importantly, the synergistic therapy of PTT and PDT has great prospect for tumor elimination. However, PTAs and PSs with high-performance are still scarce.

PTAs and PSs are the key factor to determine the effect of tumor therapy [12]. To date, a series of inorganic materials have been synthesized for anti-tumor. However, the potential cytotoxicity of inorganic materials largely restricts their further application [13]. Compared with inorganic materials, organic molecules have more unique advantages in the practice of anti-tumor therapy, such as better biocompatibility, more finely adjusted optical properties and chemical structure [14]. Therefore, organic materials are the best candidates for large-scale clinical application in the future, which has attracted more and more researchers’ attention. Currently, the U.S. Food and Drug Administration (FDA) has approved indocyanine green (ICG) for clinical application, which is a typical example [15]. However, due to ICG inferior photothermal conversion efficiency and optical instability, the application of ICG is limited obviously. In recent years, the fused-ring molecule acceptor–donor acceptor donor–acceptor (A-DA′D-A) and acceptor–donor-acceptor (A-D-A) type of organic materials, which has a narrow band-gap and strong light absorption, showed excellent performance in PTT and PDT of tumors [16–20]. For example, in 2019, Lee and co-workers found that a small molecule (F8iC) with A-D-A structure had high photothermal conversion efficiency (82%) [18]. This shows that F8iC with fused-ring structure has excellent anti-tumor effect. In 2022, another small molecule (Y16) synthesized by Yuan and co-workers also has A-DA′D-A structure, which kills tumor cells through photothermal effect and the production of toxic ROS [19]. Recently, Huang et al. chose an A-DA'D-A molecule AS1 with multifunctional phototheranostics for PTT/PDT of tumors [20]. Although modifying the alkyl chains and substituents of A-D-A and A-DA′D-A molecule can increase the number of PSs and PTAs to a certain extent, this synthesis is time-consumptive and expensive. At present, the types and quantities of organic molecular PSs and PTAs are still limited. Therefore, it is necessary to develop more molecular design strategies to construct the novel types of PSs and PTAs. However, the design of new materials for PTT/PDT of tumors by small molecular polymerization has not been reported yet, and its anti-tumor effect is still unclear. Moreover, the cell apoptosis mechanism induced by PDT and PTT is also elusive.

In order to verify the feasibility of polymerized small molecule strategy for PTT/PDT of tumor, in this work, a small molecule Y5 with A-DA′D-A structure was used as the skeleton, and thiophene unit was copolymerization to obtain PYT. To allow for the hydrophobic characteristic of polymer PYT and to endow its biocompatibility and biostability for biological applications, the corresponding PYT nanoparticles (PYT NPs) were prepared via a one-step nanoprecipitation strategy with 1,2-distearoyl-sn-glycero-3-phosphoethanolamine-N-[succinyl(polyethyleneglycol)-_2000_] (DSPE-PEG_2000_) [21–24]. PYT NPs showed excellent biosafety, ROS production capacity and photothermal conversion performance (PCE) of 67%. The effect of photothermal and ROS generated by PYT NPs on tumor cells was evaluated in vitro. As a result, the photothermal effect of PYT NPs + near infrared (NIR) and ROS killed mouse breast tumor cells (4T1), human cervical cancer cells (HeLa) and murine melanoma cells (B16F10), and the cause of cell apoptosis was related to mitochondrial damage. In vivo experiments indicated that PYT NPs had excellent tumor killing effect. We also examined the blood routine and the sections of kidney, spleen, lung, liver and heart of the mice after PYT NPs + NIR treatment, which showed that the PYT NPs + NIR group had no effect on the physiological and biochemical indexes of mice. In contrast, PYT NPs + NIR had strong PTT and PDT for tumor treatment. Therefore, the polymerized small molecule strategy is feasible in the design of anti-tumor drugs. More significantly, the variety and quantity of PTAs and PSs can be greatly increased by small molecule polymerization.

## Results and discussion

The synthetic route of the polymer PYT was shown in Scheme 1, PYT was synthesized through a simple one-step palladium-catalyzed Stille cross-coupling polymerization between Y5 and T. Structures of PYT molecule was confirmed by ^1^H NMR (Additional file 1: Fig. S1). As shown in Additional file 1: Fig. S2, PYT exhibited a number average molecular weight (*M*n) of 10.3 kDa with a polydispersity index (PDI) of 2.7, measured by high temperature gel permeation chromatography with 1,2,4-trichlorobenzene (TCB) as the eluent and polystyrene as a standard at 150 °C.

**Fig. 1:**
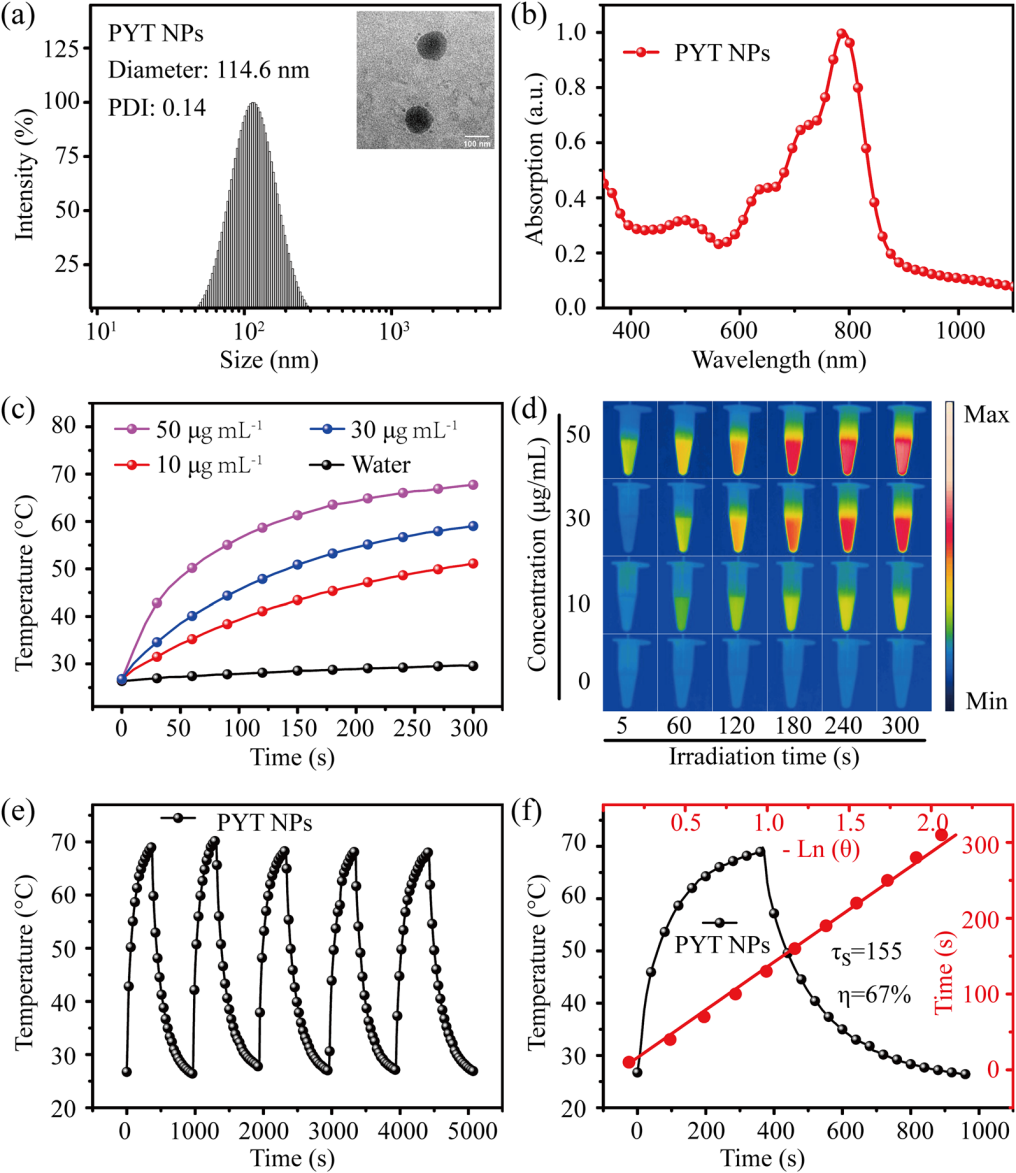
**a** DLS of PYT NPs. Inset: their TEM image. **b** UV–Vis-NIR spectrum of PYT NPs. **c** Photothermal conversion of PYT NPs at different concentrations with 808 nm laser irradiation (1.0 W cm^−2^). **d** Photothermal images of PYT NPs at various concentrations (0, 10, 30, and 50 µg mL^−1^). **e** Photothermal stability during five heating–cooling cycles. **f** The PCE (η) calculation of PYT NPs

In order to improve the biocompatibility and water solubility for biological application, hydrophobic PYT was directly encapsulated into amphiphilic block copolymer DSPE-PEG_2000_ by a one-step nanoprecipitation method to prepare PYT NPs [25, 26], as shown in Scheme 1. In PYT NPs, DSPE-PEG_2000_ self-assembled to the outer sphere of PYT NPs, and PYT built the inner core of PYT NPs. In Fig. 1a, the PYT NPs displayed a uniform spherical morphology with an average diameter of 114.6 nm, PDI value of 0.14, as confirmed by dynamic light scattering (DLS) and transmission electron microscopy (TEM), which enables PYT NPs to passively accumulate at the tumor sites via enhanced permeability and retention (EPR) effect [27]. The stability of PYT NPs after long-term storage was further examined. The diameter of PYT NPs was slightly increased during 13 days′ storage in water (Additional file 1: Fig. S3a), acidic phosphate buffer saline (PBS) (Additional file 1: Fig. S3b), PBS (Additional file 1: Fig. S3c) and serum (Additional file 1: Fig. S3d), indicating its good stability. In Fig. 1b, the ultraviolet–visible near infrared spectrum (UV–Vis-NIR) showed that PYT NPs was suitable for 808 nm laser irradiation in tumor therapy because of its maximum absorption peak at 875 nm, which is more red-shifted than ICG.

To evaluate the photothermal effect of PYT NPs [28], the temperature changes of solutions at various concentrations (10, 30, 50 μg mL^−1^) were recorded under 808 nm laser irradiation (1.0 W cm^−2^). As shwon in Fig. 1c and Fig. 1d, the temperature increased sharply along with the increasing concentration of PYT NPs, especially after 5 min laser irradiation, the temperature of PYT NPs (50 μg mL^−1^) showed an increase from 26.1 to 69.1 °C. The sharp increase in temperature with increasing concentration in a photothermal effect may be attributed to the greater absorption of photons and more efficient energy transfer within the material [29, 30]. The temperature changes of PYT NPs (50 μg mL^−1^) also were recorded under 808 nm laser irradiation with different laser powers (0.1, 0.4, 0.7, 1.0 W cm^−2^), the temperature of PYT NPs were increased to 28.6, 38.9, 48.7, 69.1 °C, respectively (Additional file 1: Fig. S4). The photothermal stability was also examined after five repeated ON/OFF cycles (Fig. 1e) under 808 nm laser irradiation (1.0 W cm^−2^), and the temperature change pattern of PYT NPs solution (50 μg mL^−1^) displayed no obvious difference. The absorption intensity of PYT NPs was maintained at 97% after 9 min laser irradiation (Additional file 1: Fig. S5). According to the previous calculation formula [31, 32], the PCE of PYT NPs was 67% (Fig. 1f). These results suggest that PYT NPs has high potential for PTT of tumor.

**Fig. 2:**
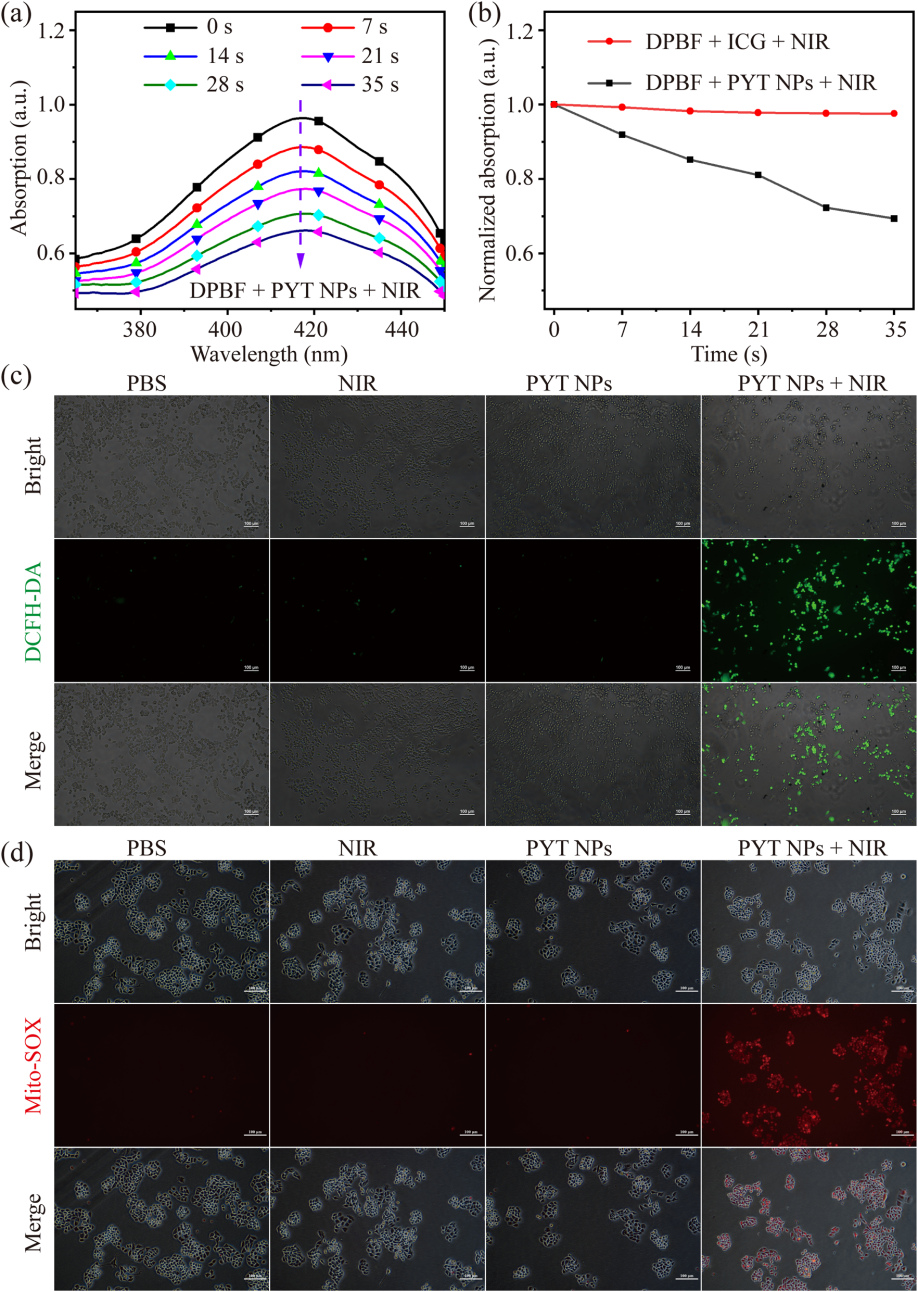
**a** ROS generation of PYT NPs mixed with DPBF under laser power density (808 nm laser, 1.0 W cm^−2^). **b** The absorption intensity of DPBF mixed with ICG or PYT NPs solution at 415 nm. **c** The images of intracellular ROS and **d** mitochondrial ROS detected in 4T1 cells treated with PBS, NIR, PYT NPS or PYT NPs + NIR. Scale bar: 100 μm

The ROS generation ability of the PYT NPs was also studied. We used 1,3-diphenylisobenzofuran (DPBF) as the indicator of total ROS [33, 34], since its absorption intensity at 415 nm is negatively related to generation of ROS. After PYT NPs co-mixing with DPBF, the absorbance at 415 nm of the solution was recorded after each 7 s-irradiation under 808 nm laser (1.0 W cm^−2^). It was found that the absorption intensity of solution decreased rapidly with irradiation time extending to 35 s (Fig. 2a). Conversely, when DPBF indicator incubated with ICG at the same concentration, the absorption intensity decreases slightly (Additional file 1: Fig. S6). The results showed that PYT NPs + NIR had a greater ROS production ability than ICG (Fig. 2b). Due to multiple D-A structures in PYT, construction of D-A structure is an effective strategy to reduce the energy gap between singlet and triplet (∆EST), which is the key factor for efficient ROS generation according to perturbation theory [35]. In addition, the photostability of PYT NPs and ICG was also studied [36, 37]. After irradiation at 808 nm (1.0 w cm^−2^) for five repeated ON/OFF cycles, the solution color of PYT NPs was not changed, which is consistent with the result of Fig. 1e, but the color of ICG solution was markedly changed (Additional file 1: Fig. S7). The results suggest PYT NPs has better photostability than ICG. The main reason of PYT NPs with higher photostability may be due to the formation of multiple O⋯S noncovalent interaction between PYT intermolecular or intramolecular [38], however, this phenomenon does not exist in ICG.

Next, the ROS generation from PYT NPs was further evaluated under the fluorescent inverted microscope. 2,7-dichlorodi-hydrofluorescein diacetate (DCFH-DA) was used to detect intracellular ROS level [39–41]. As shown in Fig. 2c, the fluorescence signal of DCF was extremely weak in cells treated with PBS, PYT NPs and NIR groups, while green fluorescence signal of DCF was much stronger in PYT NPs + NIR group, indicating that PYT NPs + NIR can effectively generate ROS. Because mitochondria are the center of cell energy metabolism, ROS and photothermal effects may affect the function of mitochondria. To further detect the level of ROS in mitochondria, the MitoSOX™ Red (Mito-SOX) was used as the indicator [42, 43]. Consistent with Fig. 2c, it was found that the red fluorescence of Mito-SOX was much stronger in 4T1 cells treated with PYT NPs + NIR (Fig. 2d), suggesting the higher mitochondrial ROS level.

In view of its outstanding photothermal conversion performance and the ability to produce toxic ROS, the phototherapy ability of PYT NPs were studied in vitro. Considering that the effective uptake of PYT NPs by cells has a decisive influence on the therapeutic effect of PTT/PDT, the uptake of PYT NPs by cancer cells was firstly examined. Although PYT had obvious fluorescence signals as shown in Fig. 6a, the maximum detection wavelength of the fluorescent inverted microscope is 660 nm which is less than the emission wavelength of PYT NPs. Thus, fluorescein isothiocyanate (FITC) labeled DSPE-PEG_2000_ was introduced into PYT NPs for detection of its cellular uptake under fluorescence microscope. The molar ratio of DSPE-PEG_2000_-FITC to DSPE-PEG_2000_ is 1:9 [44], the mixture was assembled with hydrophobic PYT by nanoprecipitation method. The uptake of FITC-labeled PYT NPs (F-PYT NPs) in 4T1 cells at 3, 6, 9, 12, 24 h after incubation was examined. As shown in Additional file 1: Fig. S8, the bright green fluorescence of FITC was observed in 4T1 cells from 6 h- to 24-incubation, suggesting that F-PYT NPs could be efficiently internalized into cancer cells and provide a prerequisite for PTT/PDT of PYT NPs.

**Fig. 3:**
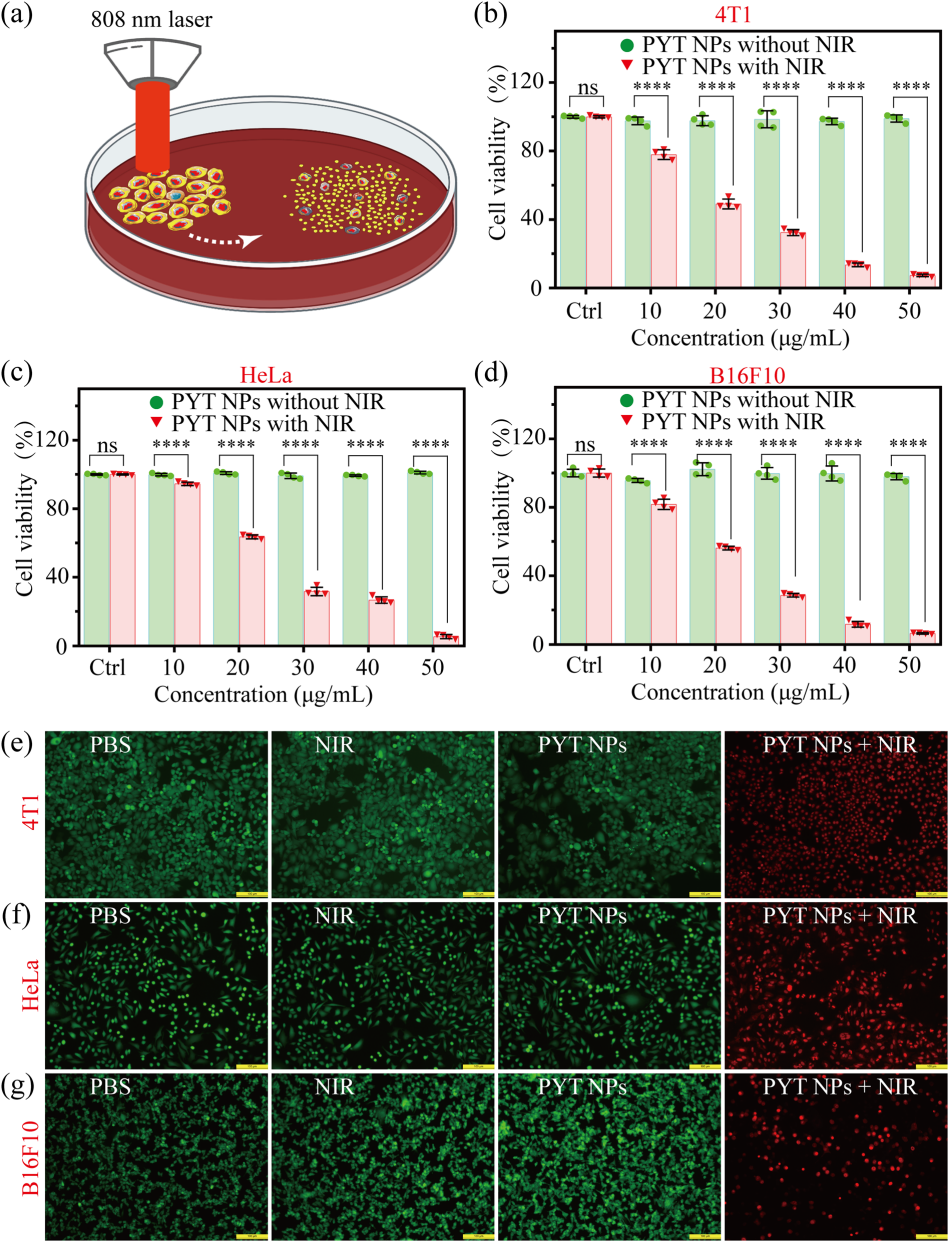
**a** Schematic diagram of PTT/PDT process. Cell viability of **b** 4T1 cells, **c** HeLa cells and **d** B16F10 cells incubated with different concentrations of PYT NPs with and without 808 nm (1.0 W cm^−2^) laser irradiation for 5 min. **b**–**d** n = 4, “ns” means “no significance”. ****P < 0.0001, *t*-test. Calcein AM (green)/propidium iodide (red) double-staining fluorescence images of **e** 4T1, **f** HeLa cells and **g** B16F10 cells treated with PBS, NIR, PYT NPs, and PYT NPs + NIR. Scale bar: 100 μm

Then, the phototherapeutic effect of PYT NPs on tumor cells was assessed with standard cell counting kit-8 (CCK-8) assays (Fig. 3a) in vitro [45, 46]. In order to investigate the biological safety of PYT NPs, dark toxicity experiments of PYT NPs (at 0–50 μg mL^−1^) for three tumor cell lines, including 4T1, HeLa and B16F10, were investigated (Additional file 1: Fig. S9). In addition, the cytotoxicity of the PYT NPs to normal cell lines, such as rat cardiomyocytes cells (H9c2), mouse embryonic fibroblast cells (NIH 3T3) and human embryonic kidney 293T cells (HEK 293T), had been examined (Additional file 1: Fig. S10). It was found PYT NPs had no cytotoxic effect on both normal cells and tumor cells. Next, the effect of PYT NPs + NIR on three tumor cell lines was assessed. Irradiation under 808 nm laser (1.0 W cm^−2^) significantly decreased viability of 4T1 (Fig. 3b), HeLa (Fig. 3c) and B16F10 (Fig. 3d) cells incubated with 10–50 μg mL^−1^ of PYT NPs, and the best therapeutic concentration of PYT NPs was 50 μg mL^−1^. Furthermore, we used the lower parameters of the laser for photothermal therapy of 4T1, HeLa and B16F10 cells. The results showed that 0.1 and 0.4 W cm^−2^ irradiation had no significant effect on cell viability, while 0.7 W and 1.0 cm^−2^ irradiation significantly decreased the viability of 4T1, HeLa and B16F10 cells (Additional file 1: Fig. S11). In addition, the best therapeutic power was 1.0 W cm^−2^. Meanwhile, the effect of PYT NPs + NIR on survival of cancer cells was further verified by double staining of Calcein-AM/PI (live/dead cell staining) [47–49]. As shown in Additional file 1: Fig. S12–S14 and Fig. 3e–g, PI positive cells was much more in 4T1, HeLa and B16F10 treated with PYT NPs + NIR than that in the cells treated with PBS, NIR or PYT NPs. In addition, scratch experiment was used to determine the effect of PYT NPs + NIR on migration of 4T1 cells. The results showed that the cell healing in PYT NPs + NIR treatment group was almost unchanged, but the cell healing in PBS, NIR or PYT NPs treatment groups was much better (Additional file 1: Fig. S15). Thus, all these data indicate that PYT NPs + NIR exerts a significantly inhibitory effect on viability, survival and migration of cancer cells.

**Fig. 4:**
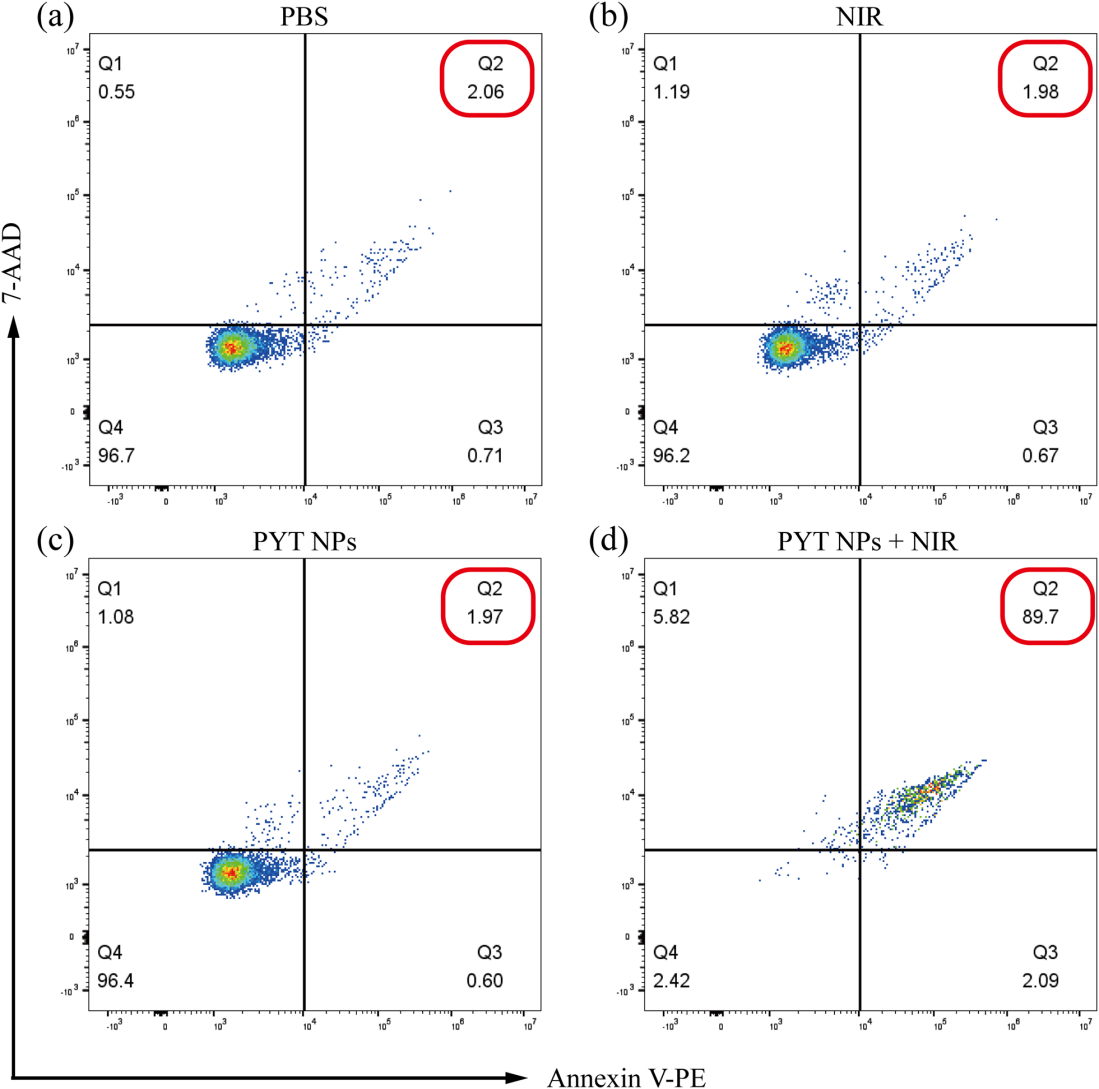
The results of flow cytometry were obtained from 4T1 cells stained with Annexin V-FITC/7-AAD after treatment with PBS, NIR, PYT NPs or PYT NPs + NIR, respectively

The phototoxicity of PYT NPs + NIR was further evaluated in 4T1 cells stained with Annexin V-fluorescein isothiocyanate (Annexin V-FITC)/7-aminoactinomycin D (7-AAD) using flow cytometry [50–52]. The Q2 and Q3 represented to late apoptosis cells and the early apoptotic cells, respectively. 4T1 cells were divided into PBS, PYT NPs, NIR and PYT NPs + NIR four groups. In Fig. 4, the apoptosis percentage in PBS treatment group was 2.77% (Fig. 4a), NIR treatment group was 2.65% (Fig. 4b), and PYT NPs group was 2.57% (Fig. 4c). However, the apoptosis percentage in PYT NPs + NIR group reached 91.79% (Fig. 4d), and 5.82% of the dead cells were distributed in the Q1, 2.42% of the alive cells were distributed in the Q4. The data of flow cytometry showed that PYT NPs + NIR had excellent cell killing ability, which is consistent with the results of CCK-8 and live/dead cell assay.

**Fig. 5:**
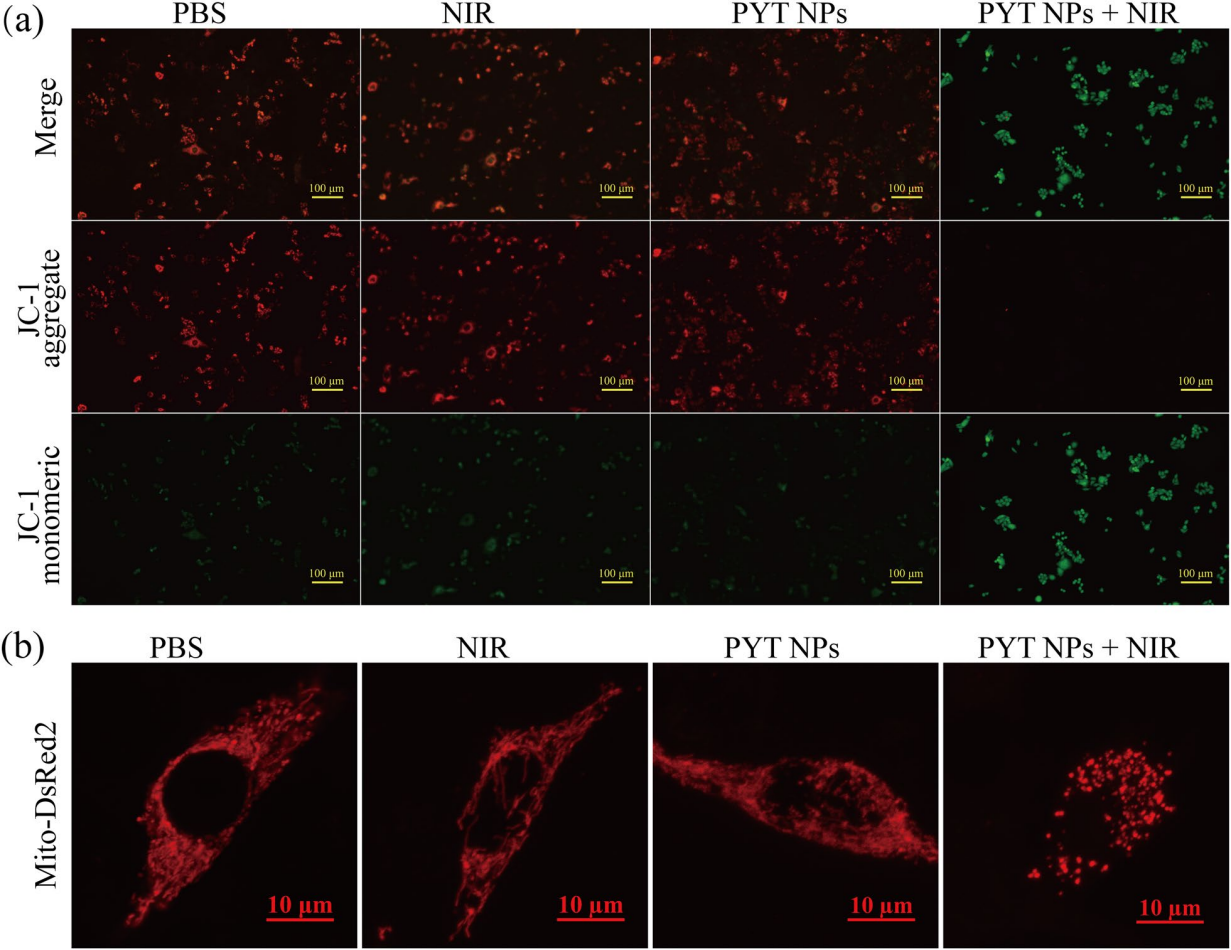
**a** Fluorescent microscope images of 4T1 cells stained with JC-1 and treated with PBS, NIR, PYT NPs, and PYT NPs + NIR. Scale bar: 100 μm. **b** Mitochondrial network morphology in 4T1 cells after different treatments. Scale bar: 10 μm

Due to its crucial role in energy metabolism, mitochondria are involved in various cellular activities, including apoptosis, differentiation, and proliferation, etc. To investigate the tumor killing mechanism of PYT NPs + NIR, mitochondrial morphology and function in cancer cells were examined. Firstly, the expression of pro-apoptotic protein (Bax) and anti-apoptotic protein (Bcl-2) related to mitochondria were detected using western blotting assay [53–55]. Compared to PBS, NIR and PYT groups, PYT NPs + NIR upregulated the expression of Bax and decreased Bcl-2 level in 4T1 cells (Additional file 1: Fig. S16). Bax is a pro-apoptotic protein with multiple Bcl-2 homologous domains, upregulation of Bax can promote the opening of mitochondrial permeability transition pore (mPTP), and disrupt mitochondrial membrane potential (MMP). Then, 5,5′,6,6′-Tetrachloro-1,1′,3,3′-tetraethyl-imidacarbocyanine (JC-1) was used as an indicator of MMP [56, 57]. A sharp increase in green fluorescence of JC-1 monomer and decrease in red fluorescence of JC-1 aggregate were detected in 4T1 cells treated with PYT NPs + NIR (Fig. 5a), suggesting a decrease of MMP level and damage in mitochondrial function. In order to observe the morphological changes of mitochondria, 4T1 cells were transfected with mito-DsRed2 plasmid [58, 59]. As shown in Fig. 5b, mitochondria in 4T1 cells treated with PBS, NIR or PYT NPs display as the thread-like or tubular structures under laser confocal microscope. However, the morphology of mitochondrial changed into dot-like structures or fragmented network in PYT NPs + NIR group. The above results suggest that the anti-tumor effect of PYT NPs + NIR is related to mitochondrial apoptosis pathway [60, 61].

**Fig. 6:**
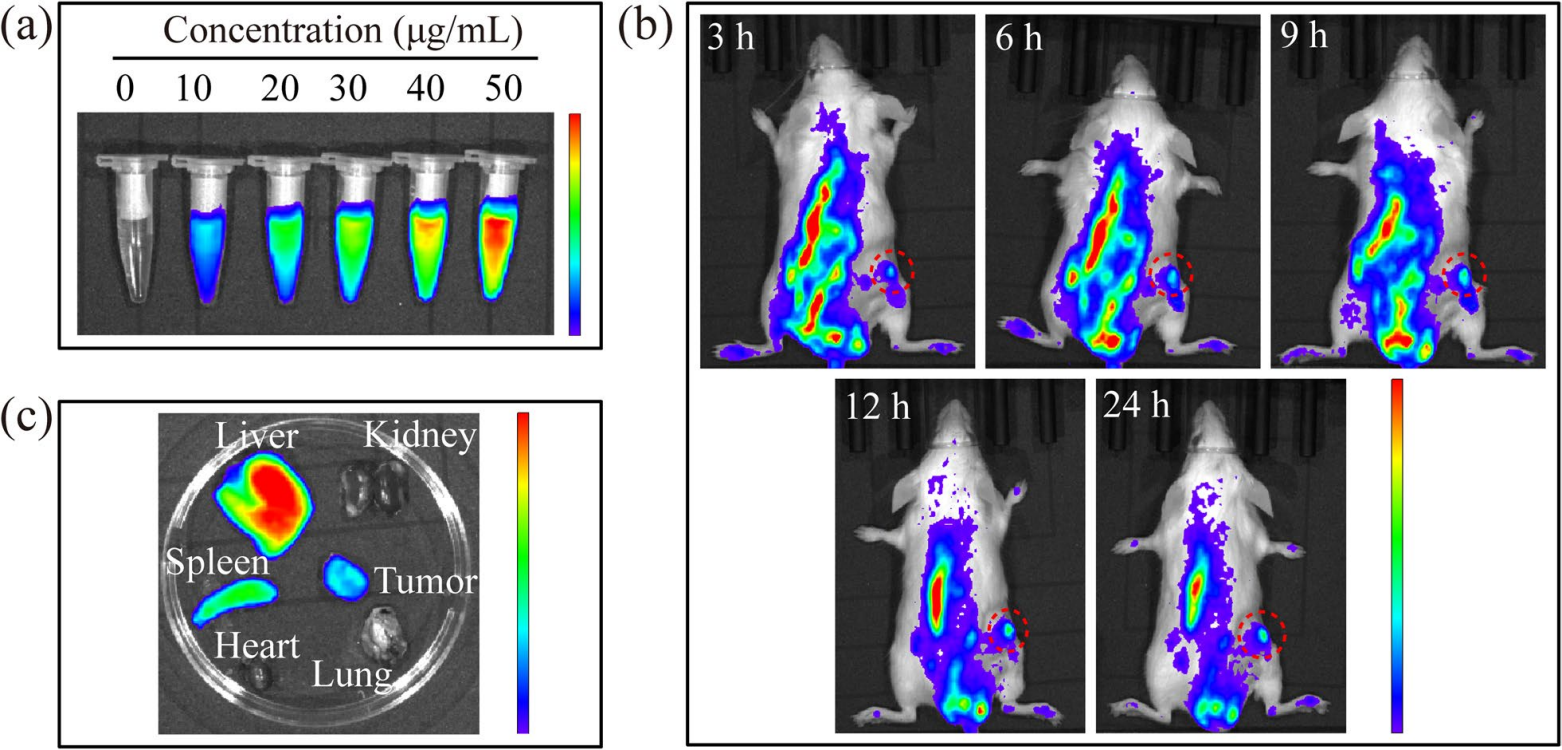
**a** In vitro fluorescence intensity of PYT NPs at different concentrations. **b** In vivo fluorescence images of mouse at different time points after injection (4T1 tumor bearing mice) **c** Ex vivo fluorescence image of tumors and major organs

Due to its excellent PTT/PDT efficiency in vitro, the anti-tumor effect of PYT NPs + NIR was also studied in vivo. Firstly, the blood compatibility of PYT NPs was assessed in vitro [62–64]. PYT NPs at different concentrations (0, 0.25, 0.50, 0.75, 1.00 mg mL^−1^) were mixed with red blood cells from BALB/c mice. It was found that the hemolysis rates were all less than 5%, indicating the good blood compatibility of PYT NPs (Additional file 1: Fig. S17). Subsequently, antitumor efficacy of PYT NPs in vivo were evaluated in 4T1 tumor bearing BALB/c mice [65, 66]. To explore the best phototherapy time, fluorescence imaging of PYT NPs was studied in vitro. The fluorescence intensity of PYT NPs exhibited a concentration-dependent manner (Fig. 6a). The fluorescence properties of PYT NPs are suitable for tumor molecular imaging in vivo. In this study, the PYT NPs were injected into 4T1 tumor bearing mice through the tail vein and the fluorescence imaging of mice were collected at 3, 6, 9, 12 and 24 h after injection. The PYT NPs could passively target the tumor via EPR effects. The fluorescence of tumor reached the highest intensity at 12 h after injection (Fig. 6b), which proved that PYT NPs had good tumor enrichment ability and suggest the best time for phototherapy. Next, the mice were sacrificed to evaluate the ex vivo biodistribution of PYT NPs. It was observed that the liver and spleen exhibited the strongest fluorescence signals, followed by the tumor (Fig. 6c). As time extended to 24 h, the fluorescence intensity of tumor, spleen and liver decreased (Fig. 6b), indicating that PYT NPs can be metabolized by the hepatobiliary system of mice. Thus, these results show that PYT NPs can be used as a potential agent for tumor imaging.

At the same time, photothermal imaging was also used to explore the best treatment time for tumors. The temperature changes in tumors were monitored by infrared thermal imaging. At the beginning, the tumor temperature was almost unchanged after laser irradiation (808 nm, 1.0 W cm^−2^) because the PYT NPs had not been delivered to the tumor through blood circulation. As the time extended, the temperature of tumor was increased from 36.4 °C to 59.6 °C by laser irradiation at 12 h (Additional file 1: Fig. S18). Compared to 12 h after injection, the laser irradiation-induced tumor temperature was reduced at 24 h, which may be due to the metabolism of PYT NPs. Thus, the best time point for PTT/PDT of tumor was at 12 h after PYT NPs injection.

**Fig. 7:**
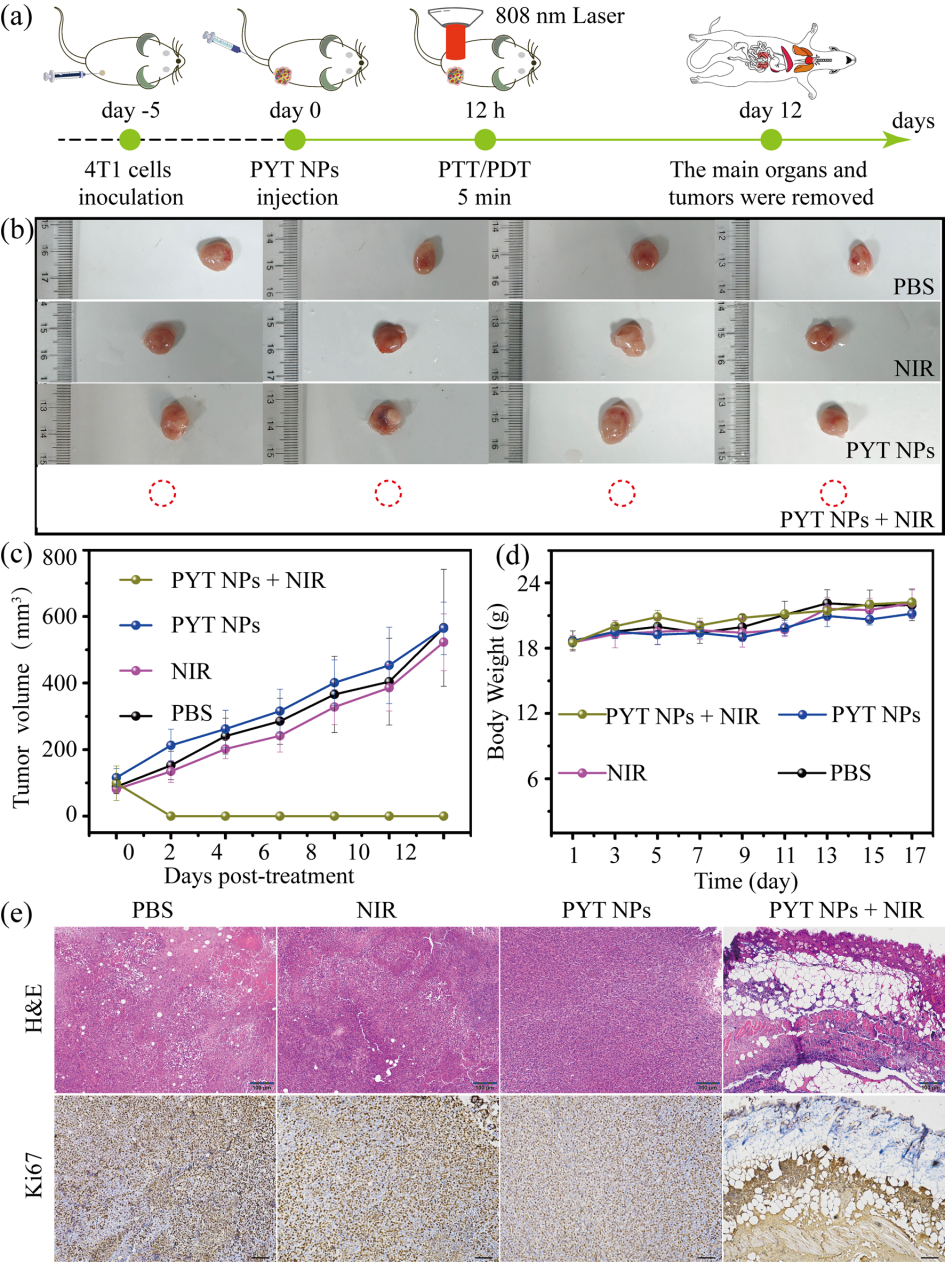
**a** Schematic diagram of PYT NPs + NIR for the tumor therapy. **b** Images of tumors removed from BALB/c mice in PBS, PYT NPs, NIR and PYT NPs + NIR groups. **c** Tumor growth curves and **d** changes in body weights of 4T1 tumor-bearing mice. **e** The representative images of H&E and Ki67 staining of tissue sections from mice treated with PBS, NIR, PYT NPs, or PYT NPs + NIR, respectively. Scale bar: 100 μm

The good performance of PTT and PDT in vitro as well as efficient tumor accumulation ability of PYT NPs encouraged us to evaluate its anti-tumor efficacy in vivo. The detail schematic diagram of PTT/PDT process is shown in Fig. 7a. Mice bearing 4T1 tumor were randomly divided into PBS, PYT NPs, NIR and PYT NPs + NIR (four mice per group) (Additional file 1: Fig. S19). In Fig. 7b and Fig. 7c, the tumor was completely disappeared in mice treated with PYT NPs + NIR. On the contrary, the tumors showed almost the same growth trend in the mice treated with PBS, NIR and PYT NPs, as the tumor volume increased without significant difference among the groups (Fig. 7c). During the treatment, there was no significant difference in body weight of mice in different groups (Fig. 7d). In addition, compared with the PBS, PYT NPs, NIR groups, the results of hematoxylin–eosin (H&E) and Ki67 staining (H&E and Ki67 staining method refers to previous reports [67–71]) showed that the tumor tissue in PYT NPs + NIR group disappeared at 12 days after irradiation, only connective tissue and granulation tissue were observed in irradiation region (Fig. 7e). Therefore, these in vivo results demonstrate PYT NPs + NIR may be a potent strategy for PTT/PDT of tumors in the future.

**Fig. 8:**
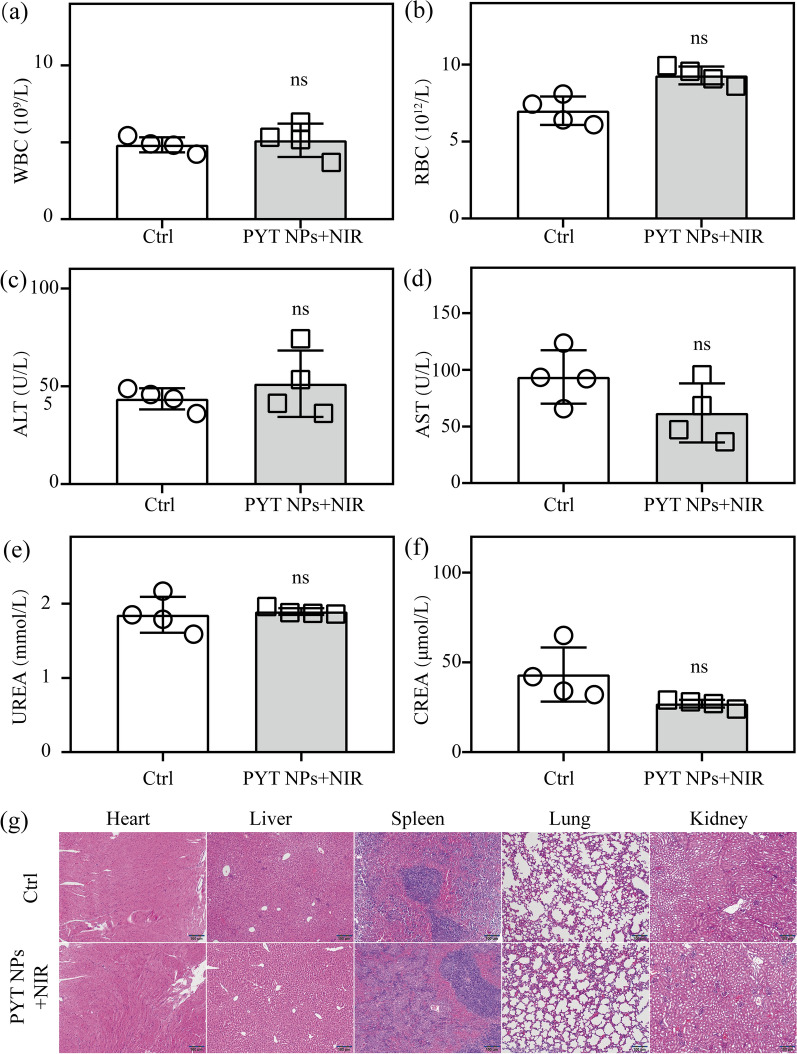
Routine blood indexes of **a** WBC, **b** RBC, hepatic function markers of **c** ALT, **d** AST, as well as renal function markers of **e** CREA, **f** UREA obtained by control mice and PYT NPs + NIR treated mice, respectively. **a**–**f** n = 4, “ns” means “no significance”. ****P < 0.0001, *t*-test. **g** H&E staining of major organs (heart, liver, spleen, lung, kidney) collected from control mice (saline) and mice treated with PYT NPs + NIR. Scale bar: 100 μm

Moreover, the biocompatibility of PYT NPs is important for in vivo experiments, especially for clinical translation in the future [72–75]. The blood was collected for blood routine test (Additional file 1: Table S1) and serum biochemical indexes (Additional file 1: Table S2) before removing the vital organs of mice. As shown in Fig. 8a–f, white blood cell (WBC) and red blood cell (RBC) counts are within the normal range. There was no statistical difference in serum biochemical indexes between control mice (saline) and PYT NPs + NIR treated mice, including alanine aminotransferase (ALT), aspartate aminotransferase (AST), creatinine (CREA) and urea (UREA). Furthermore, histopathological examination of vital organs of mice (kidney, spleen, lung, liver and heart) was conducted. H&E staining of these tissue slices showed that the structure of each organ is clear and complete, and the cell morphology is normal (Fig. 8g). The myocardial fibers of cardiac cells are pink, with clear nucleus structure and no obvious lesions. The structure of hepatic lobule is clear and complete, and the hepatic cord is empty. There is no obvious abnormality in white pulp, red pulp and marginal zone of spleen. Alveolar structure is complete, alveolar cavity is empty, and no abnormal changes are found in pulmonary interstitium. The normal structure of glomerulus is clear, the outline of renal balloon is complete and there is no obvious damage. These results of blood routine test, serum biochemical indexes and H&E staining demonstrate that PYT NPs can be used as a therapeutic agent with good biocompatibility for PTT/PDT of tumor.

## Conclusions

In conclusion, PTT and PDT are an effective method for tumor treatment. We used Y5 molecule to polymerize with thiophene unit to obtain polymer PYT, the corresponding PYT NPs were prepared according to a one-step nanoprecipitation strategy with DSPE-PEG_2000_. PYT NPs + NIR was demonstrated to have excellent photothermal conversion performance and ROS-production capacity, which are the premise of PTT and PDT for tumors. In vitro and *vivo* experiments indicate that PYT NPs + NIR had outstanding tumor killing effect, and irradiation-mediated apoptosis was due to mitochondrial damage. In this study, the polymerized small molecule strategy is feasible in the design of anti-tumor drugs. Especially, the variety and quantity of PTAs and PSs can be increased by small molecule polymerization, which will greatly improve the performance of PTT/PDT for tumor.

### Supplementary Information

**Additional file 1:** **Figure S1.** ^1^H NMR spectrum of PYT in CDCl3. **Figure S2.** GPC spectra of PYT in TCB, estimated by high temperature gel permeation chromatography with 150 °C. **Figure S3.** Diameters of PYT NPs after storage in **a** water, **b** acidic PBS, **c** PBS and **d** serum for different times. **Figure S4.** The temperature changes of PYT NPs (50 μg mL^−1^) were recorded under 808 nm laser irradiation with different laser powers. **Figure S5.** UV–Vis-NIR spectra of PYT NPs during continuous 808 nm laser irradiation (1.0 W cm^−2^) for 0, 3,6, 9 min, respectively. **Figure S6.** Time-related ROS generation of ICG mixed with DPBF under laser power density (808 nm laser, 1.0 W cm^−2^). **Figure S7.** The photographs of PYT NPs (right) and ICG (left) before and after five repeated ON/OFF cycles irradiation with 808 nm lasers (1.0 W cm^−2^), respectively. **Figure S8.** Cellular uptake efficiency of FITC-labeled PYT NPs in 4T1 cells. Scale bar: 100 μm. **Figure S9.** Cell viability of **a** 4T1, **b** B16F10 and **c** HeLa after treatment with various concentrations of PYT NPs under dark condition for 48 h. “ns” means “no significance”, one-way ANOVA. **Figure S10.** Cell viability of **a** H9c2, **b** NIH 3T3 and **c** HEK 293 T after treatment with various concentrations of PYT NPs under dark condition for 48 h. n = 4, “ns” means “no significance”, one-way ANOVA. **Figure S11.** Cell viability of **a** 4T1, **b** HeLa and **c** B16F10 after treatment with different laser power values (808 nm). n = 4, “ns” means “no significance”, *P*-value: ****P < 0.0001, one-way ANOVA. **Figure S12.** Live/dead staining of 4T1 cancer cells after different treatments. Scale bar: 100 μm. **Figure S13.** Live/dead staining of Hela cancer cells after different treatments. Scale bar: 100 μm. **Figure S14.** Live/dead staining of B16F10 cancer cells after different treatments. Scale bar: 100 μm. **Figure S15.** In vitro wound scratch assay of 4T1 cells with different treatments. Scale bar: 100 μm. **Figure S16.** Expression of Bcl-2 and Bax in 4T1 cells incubated with PBS or PYT NPS (50 μg mL^−1^ for PYT NPs) was detected by western blot analysis. β-actin was used as the internal control. **Figure S17.** **a** Hemolytic effect of PYT NPs at different concentrations on RBCs from BALB/c mice, water group was used as the positive control, and PBS as the negative control, respectively. **b** Representative photos of hemolysis experiment results. n = 3, “ns” means “no significance”,* P*-value: ****P < 0.0001. **Figure S18.** **a** Infrared images were collected from 4T1 tumor-bearing BALB/c mice at different time intervals after 808 nm laser irradiation. **b** Taking temperature as the ordinate and time as the abscissa, the temperature curve of laser-heated mouse tumor site was drawn. n = 3, ****P < 0.0001, one-way ANOVA. **Figure S19.** Representative pictures of photothermal therapy for 4T1 tumor bearing mice after different treatments (PBS, NIR, PYT NPs and PYT NPs + NIR) in vivo. **Table S1.** Hepatic function indexes (ALB, ALT and AST) and renal function markers (UREA, CREA and UA) of mice at day 12 after treatment. **Table S2.** Routine blood indexes of mice at day 12 after treatment.

## Data Availability

The raw data and processed data required to reproduce these findings are available from the corresponding author upon request.

## References

[1] Siegel RL, Miller KD, Wagle NS, Jemal A (2023). Cancer statistics, 2023. CA Cancer J Clin.

[2] Feng R, Su Q, Huang X, Basnet T, Xu X, Ye W (2023). Cancer situation in China: what does the China cancer map indicate from the first national death survey to the latest cancer registration?. Cancer Commun.

[3] Zhang C, Chen W, Zhang T, Jiang X, Hu Y (2020). Hybrid nanoparticle composites applied to photodynamic therapy: strategies and applications. J Mater Chem B.

[4] Li J, Kang M, Zhang Z, Li X, Xu W, Wang D, Gao X, Tang BZ (2023). Synchronously manipulating absorption and extinction coefficient of semiconducting polymers via precise dual-acceptor engineering for NIR-II excited photothermal theranostics. Angew Chem Int Ed.

[5] Zhang C, Yuan Y, Wu K, Wang Y, Zhu S, Shi J, Wang L, Li Q, Zuo X, Fan C, Chang C, Li J (2022). Driving DNA origami assembly with a terahertz wave. Nano Lett.

[6] Teng KX, Chen WK, Niu LY, Fang WH, Cui G, Yang QZ (2021). BODIPY-based photodynamic agents for exclusively generating superoxide radical over singlet oxygen. Angew Chem Int Ed.

[7] Yang Z, Zhang Z, Sun Y, Lei Z, Wang D, Ma H, Tang BZ (2021). Incorporating spin-orbit coupling promoted functional group into an enhanced electron D-A system: A useful designing concept for fabricating efficient photosensitizer and imaging-guided photodynamic therapy. Biomaterials.

[8] Zhang Z, Ding D, Liu J, Huang C, Li W, Lu K, Cheng N (2023). Supramolecular nanozyme system based on polydopamine and polyoxometalate for photothermal-enhanced multienzyme cascade catalytic tumor therapy. ACS Appl Mater Interfaces.

[9] Ma G, Liu Z, Zhu C, Chen H, Kwok RTK, Zhang P, Tang BZ, Cai L, Gong P (2022). H_2_O_2_-responsive NIR-II AIE nanobomb for carbon monoxide boosting low-temperature photothermal therapy. Angew Chem Int Ed.

[10] Jiang Z, Zhang C, Wang X, Yan M, Ling Z, Chen Y, Liu Z (2021). A borondifluoride-complex-based photothermal agent with an 80 % photothermal conversion efficiency for photothermal therapy in the NIR-II window. Angew Chem Int Ed.

[11] Wu W, Shi L, Duan Y, Xu S, Shen L, Zhu T, Hou L, Meng X, Liu B (2021). Nanobody modified high-performance AIE photosensitizer nanoparticles for precise photodynamic oral cancer therapy of patient-derived tumor xenograft. Biomaterials.

[12] Gao M, Huang X, Wu Z, Wang L, Yuan S, Du Z, Luo S, Li R, Wang W (2022). Synthesis of a versatile mitochondria-targeting small molecule for cancer near-infrared fluorescent imaging and radio/photodynamic/photothermal synergistic therapies. Mater Today Bio.

[13] Shao W, Zhao F, Xue J, Huang L (2023). NIR-II absorbing organic nanoagents for photoacoustic imaging and photothermal therapy. BMEMat.

[14] Choi Y, Min K, Han N, Tae G, Kim DY (2023). Novel Application of NIR-I-absorbing quinoidal conjugated polymer as a photothermal therapeutic agent. ACS Appl Mater Interfaces.

[15] Xue D, Wang Y, Zhang H (2023). Advances of NIR light responsive materials for diagnosis and treatment of brain diseases. Adv Opt Mater.

[16] Li B, Gan Y, Yang K, Pang E, Ren X, Zhao S, He D, Zhao F, Wang B, Yin P, Song X, Lan M (2023). Acceptor-donor-acceptor structured phototheranostics for near-infrared II fluorescent and photoacoustic imaging-guided photodynamic and photothermal synergistic therapy. Sci China Mater.

[17] Zheng R, Zhao Q, Qing W, Li S, Liu Z, Li Q, Huang Y (2022). Carrier-free delivery of ultrasmall π-conjugated oligomer nanoparticles with photothermal conversion over 80% for cancer theranostics. Small.

[18] Li X, Liu L, Li S, Wan Y, Chen J-X, Tian S, Huang Z, Xiao Y-F, Cui X, Xiang C, Tan Q, Zhang X-H, Guo W, Liang X-J, Lee C-S (2019). Biodegradable π-conjugated oligomer nanoparticles with high photothermal conversion efficiency for cancer theranostics. ACS Nano.

[19] Yang K, Long F, Liu W, Zhang Z, Zhao S, Wang B, Zou Y, Lan M, Yuan J, Song X, Lin C (2022). A-DA'D-A structured organic phototheranostics for NIR-II fluorescence/photoacoustic imaging-guided photothermal and photodynamic synergistic therapy. ACS Appl Mater Interfaces.

[20] Li M, Lu Z, Zhang J, Chen L, Tang X, Jiang Q, Hu Q, Li L, Liu J, Huang W (2023). Near-infrared-II fluorophore with inverted dependence of fluorescence quantum yield on polarity as potent phototheranostics for fluorescence image-guided phototherapy of tumors. Adv Mater.

[21] Zhang C, Ren J, Yang Y, Wang D, He J, Huo D, Hu Y (2016). Ultra-sensitive diagnosis of orthotopic patient derived hepatocellular carcinoma by Fe@graphene nanoparticles in MRI. RSC Adv.

[22] Guo W, Chen Z, Chen J, Feng X, Yang Y, Huang H, Liang Y, Shen G, Liang Y, Peng C, Li Y, Li G, Huang W, Zhao B, Hu Y (2020). Biodegradable hollow mesoporous organosilica nanotheranostics (HMON) for multi-mode imaging and mild photo-therapeutic-induced mitochondrial damage on gastric cancer. J Nanobiotechnol.

[23] Li C, Jiang G, Yu J, Ji W, Liu L, Zhang P, Du J, Zhan C, Wang J, Tang BZ (2022). Fluorination enhances NIR-II emission and photothermal conversion efficiency of phototheranostic agents for imaging-guided cancer therapy. Adv Mater.

[24] Song L, Chen B, Qin Z, Liu X, Guo Z, Lou H, Liu H, Sun W, Guo C, Li C (2022). Temperature-dependent CAT-like RGD-BPNS@SMFN nanoplatform for PTT-PDT self-synergetic tumor phototherapy. Adv Healthc Mater.

[25] Zhu Y, Lai H, Guo H, Peng D, Han L, Gu Y, Wei Z, Zhao D, Zheng N, Hu D, Xi L, He F, Tian L (2022). Side-chain-tuned molecular packing allows concurrently boosted photoacoustic imaging and NIR-II fluorescence. Angew Chem Int Ed.

[26] Wang M, Li Y, Wang M, Liu K, Hoover AR, Li M, Towner RA, Mukherjee P, Zhou F, Qu J, Chen WR (2022). Synergistic interventional photothermal therapy and immunotherapy using an iron oxide nanoplatform for the treatment of pancreatic cancer. Acta Biomater.

[27] Yang K, Zhang Z, Gan Y, Tan Q, Huang L, Wang B, Hu G, Yin P, Song X, Lan M (2022). Photovoltaic molecules with ultra-high light energy utilization for near-infrared laser triggered synergetic photodynamic and photothermal therapy. J Mater Chem B.

[28] Lin R, Liu J, Xu W, Liu Z, He X, Zheng C, Kang M, Li X, Zhang Z, Feng H-T, Lam JWY, Wang D, Chen M, Tang BZ (2023). Type I photosensitization with strong hydroxyl radical generation in NIR dye boosted by vigorous intramolecular motions for synergistic therapy. Adv Mater.

[29] Liu Y, Zhao J, Xu X, Xu Y, Cui W, Yang Y, Li J (2023). Emodin-based nanoarchitectonics with giant two-photon absorption for enhanced photodynamic therapy. Angew Chem Int Ed.

[30] Liu LC, Pan Y, Ye LY, Zhang T, Chen Y, Liang C, Chen DP, Mou XZ, Dong XC, Cai Y (2023). Space and bond synergistic conjugation controlling multiple-aniline NIR-II absorption for photoacoustic imaging guided photothermal therapy. Adv Healthc Mater.

[31] Yu J, Wang L, Xie X, Zhu W, Lei Z, Lv L, Yu H, Xu J, Ren J (2023). Multifunctional nanoparticles codelivering doxorubicin and amorphous calcium carbonate preloaded with indocyanine green for enhanced chemo-photothermal cancer therapy. Int J Nanomedicine.

[32] Yu Y, Tang D, Liu C, Zhang Q, Tang L, Lu Y, Xiao H (2022). Biodegradable polymer with effective near-infrared-II absorption as a photothermal agent for deep tumor therapy. Adv Mater.

[33] Wang Y, Dai X, Dong C, Guo W, Xu Z, Chen Y, Xiang H, Zhang R (2022). Engineering electronic band structure of binary thermoelectric nanocatalysts for augmented pyrocatalytic tumor nanotherapy. Adv Mater.

[34] Bian H, Ma D, Zhang X, Xin K, Yang Y, Peng X, Xiao Y (2021). Tailored engineering of novel xanthonium polymethine dyes for synergetic PDT and PTT triggered by 1064 nm laser toward deep-seated tumors. Small.

[35] Chen J, Wen K, Chen H, Jiang S, Wu X, Lv L, Peng A, Zhang S, Huang H (2020). Achieving high-performance photothermal and photodynamic effects upon combining D-A structure and nonplanar conformation. Small.

[36] Zhao H, Xu J, Feng C, Ren J, Bao L, Zhao Y, Tao W, Zhao Y, Yang X (2022). Tailoring aggregation extent of photosensitizers to boost phototherapy potency for eliciting systemic antitumor immunity. Adv Mater.

[37] Yin C, Tai X, Li X, Tan J, Lee C-S, Sun P, Fan Q, Huang W (2022). Side chain engineering of semiconducting polymers for improved NIR-II fluorescence imaging and photothermal therapy. Chem Eng J.

[38] Huang H, Yang L, Facchetti A, Marks TJ (2017). Organic and polymeric semiconductors enhanced by noncovalent conformational locks. Chem Rev.

[39] Zhu Z, Ma A-H, Zhang H, Lin T-Y, Xue X, Farrukh H, Zhu S, Shi W, Yuan R, Cao Z, Chittepu VCSR, Prabhala R, Li Y, Lam KS, Pan CX (2022). Phototherapy with cancer-specific nanoporphyrin potentiates immunotherapy in bladder cancer. Clin. Cancer Res..

[40] Hu X, Li H, Li R, Qiang S, Chen M, Shi S, Dong C (2023). A phase-change mediated intelligent nanoplatform for chemo/photothermal/photodynamic therapy of cancer. Adv Healthcare Mater.

[41] Wu Y, Song X, Zhou X, Song R, Tang W, Yang D, Wang Y, Lv Z, Zhong W, Cai H-L, Zhang A, Wei J, Wu XS (2023). Piezo-activated atomic-thin molybdenum disulfide/mxene nanoenzyme for integrated and efficient tumor therapy via ultrasound-triggered schottky electric field. Small.

[42] Cetin Ersen B, Goncu B, Dag A, Birlik DG (2023). GLUT-targeting phototherapeutic nanoparticles for synergistic triple combination cancer therapy. ACS Appl Mater Interfaces.

[43] Murphy MP, Bayir H, Belousov V, Chang CJ, Davies KJA, Davies MJ, Dick TP, Finkel T, Forman HJ, Janssen-Heininger Y, Gems D, Kagan VE, Kalyanaraman B, Larsson NG, Milne GL, Nystrom T, Poulsen HE, Radi R, Van Remmen H, Schumacker PT, Thornalley PJ, Toyokuni S, Winterbourn CC, Yin H, Halliwell B (2022). Guidelines for measuring reactive oxygen species and oxidative damage in cells and in vivo. Nat Metab.

[44] Yan D, Wang M, Wu Q, Niu N, Li M, Song R, Rao J, Kang M, Zhang Z, Zhou F, Wang D, Tang BZ (2022). Multimodal imaging-guided photothermal immunotherapy based on a versatile NIR-II aggregation-induced emission luminogen. Angew Chem Int Ed.

[45] Zhang G, Chen X, Chen X, Du K, Ding K, He D, Ding D, Hu R, Qin A, Tang BZ (2023). Click-reaction-mediated chemotherapy and photothermal therapy synergistically inhibit breast cancer in mice. ACS Nano.

[46] Xu W, Lu J, Guo Z, Ye J, Gao X, Li Y, Xie W, Zhao L (2022). Hypoxia alleviated and one photo-triggered thermal/dynamic nanoplatform for immunogenic cell death-initiated cancer immunotherapy. ACS Appl Bio Mater.

[47] Zhang C, Ren J, Hua J, Xia L, He J, Huo D, Hu Y (2018). Multifunctional Bi_2_WO_6_ nanoparticles for CT-guided photothermal and oxygen-free photodynamic therapy. ACS Appl Mater Interfaces.

[48] Zhao P, Ren S, Liu Y, Huang W, Zhang C, He J (2018). PL-W_18_O_49_-TPZ nanoparticles for simultaneous hypoxia-activated chemotherapy and photothermal therapy. ACS Appl Mater Interfaces.

[49] Zhang C, Jing X, Guo L, Cui C, Hou X, Zuo T, Liu J, Shi J, Liu X, Zuo X, Li J, Chang C, Fan C, Wang L (2021). Remote photothermal control of DNA origami assembly in cellular environments. Nano Lett.

[50] Torquato HFV, Rodrigues Junior MT, Lima CS, de Araujo Junior RT, Talhati F, Dias DA, Justo GZ, Ferreira AT, Pilli RA, Paredes-Gamero EJ (2022). A canthin-6-one derivative induces cell death by apoptosis/necroptosis-like with DNA damage in acute myeloid cells. Biomed Pharmacother.

[51] Zhu H, Yang C, Yan A, Qiang W, Ruan R, Ma K, Guan Y, Li J, Yu Q, Zheng H, Tu L, Liu S, Dai Z, Sun Y (2023). Tumor-targeted nano-adjuvants to synergize photomediated immunotherapy enhanced antitumor immunity. View.

[52] Zuo H, Tao J, Shi H, He J, Zhou Z, Zhang C (2018). Platelet-mimicking nanoparticles co-loaded with W_18_O_49_ and metformin alleviate tumor hypoxia for enhanced photodynamic therapy and photothermal therapy. Acta Biomater.

[53] Shi Z, Bai H, Wu J, Miao X, Gao J, Xu X, Liu Y, Jiang J, Yang J, Zhang J, Shao T, Peng B, Ma H, Zhu D, Chen G, Hu W, Li L, Huang W (2023). Acceptor engineering produces ultrafast nonradiative decay in NIR-II aza-BODIPY nanoparticles for efficient osteosarcoma photothermal therapy via concurrent apoptosis and pyroptosis. Research.

[54] Zhang T, Wu B, Akakuru OU, Yao C, Sun S, Chen L, Ren W, Wu A, Huang P (2021). Hsp90 inhibitor-loaded IR780 micelles for mitochondria-targeted mild-temperature photothermal therapy in xenograft models of human breast cancer. Cancer Lett.

[55] Cai H, Dai X, Wang X, Tan P, Gu L, Luo Q, Zheng X, Li Z, Zhu H, Zhang H, Gu Z, Gong Q, Luo K (2020). A nanostrategy for efficient imaging-guided antitumor therapy through a stimuli-responsive branched polymeric prodrug. Adv Sci.

[56] Cui X, Deng X, Liang Z, Lu J, Shao L, Wang X, Jia F, Pan Z, Hu Q, Xiao X, Wu Y, Sheng W (2021). Multicomponent-assembled nanodiamond hybrids for targeted and imaging guided triple-negative breast cancer therapy via a ternary collaborative strategy. Biomater Sci.

[57] Li ZZ, Guo LS, Lin LQ, Wang TT, Jiang YQ, Song J, Feng JH, Huang JF, Li HY, Bai ZH, Liu WQ, Zhang JF (2023). Porous SiO_2_-based reactor with self-supply of O_2_ and H_2_O_2_ for synergistic photo-thermal/photodynamic therapy. Int J Nanomed.

[58] Zou GP, Wang T, Xiao JX, Wang XY, Jiang LP, Tou FF, Chen ZP, Qu XH, Han XJ (2023). Lactate protects against oxidative stress-induced retinal degeneration by activating autophagy. Free Radical Biol Med.

[59] Gajewska KA, Lescesen H, Ramialison M, Wagstaff KM, Jans DA (2021). Nuclear transporter Importin-13 plays a key role in the oxidative stress transcriptional response. Nat Commun.

[60] Zhuang J, Wang B, Chen H, Zhang K, Li N, Zhao N, Tang BZ (2023). Efficient NIR-II type-I AIE photosensitizer for mitochondria-targeted photodynamic therapy through synergistic apoptosis-ferroptosis. ACS Nano.

[61] Guo X, Yang N, Ji W, Zhang H, Dong X, Zhou Z, Li L, Shen H-M, Yao SQ, Huang W (2021). Mito-bomb: targeting mitochondria for cancer therapy. Adv Mater.

[62] Zhao X, Zhang K-K, Chen L-J, Wang Z-Y, Yan X-P (2023). Multifunctionalized tumor-triggered targeting theranostic nanoparticles as a precision nir imaging-guided nanoplatform for photothermal/photodynamic therapy. ACS Appl Nano Mater.

[63] Li J, Wang R, Sun Y, Xiao P, Yang S, Wang X, Fan Q, Wu W, Jiang X (2021). NIR-II fluorophore with dithienylethene as an electron donor for fluorescence/photoacoustic dual-model imaging and photothermal therapy. ACS Appl Mater Interfaces.

[64] Frantellizzi V, Verrina V, Raso C, Pontico M, Petronella F, Bertana V, Ballesio A, Marasso SL, Miglietta S, Rosa P, Scibetta S, Petrozza V, De Feo MS, De Vincentis G, Calogero A, Pani R, Perotto G, De Sio L (2022). ^99m^Tc-labeled keratin gold-nanoparticles in a nephron-like microfluidic chip for photo-thermal therapy applications. Mater Today Adv.

[65] Huang H, Zhang C, Wang X, Shao J, Chen C, Li H, Ju C, He J, Gu H, Xia D (2020). Overcoming hypoxia-restrained radiotherapy using an erythrocyte-inspired and glucose-activatable platform. Nano Lett.

[66] Zhang C, Cheng X, Chen M, Sheng J, Ren J, Jiang Z, Cai J, Hu Y (2017). Fluorescence guided photothermal/photodynamic ablation of tumours using pH-responsive chlorin e6-conjugated gold nanorods. Colloids Surf B Biointerfaces.

[67] Xie H, Bi Z, Yin J, Li Z, Hu L, Zhang C, Zhang J, Lam JWY, Zhang P, Kwok RTK, Li K, Tang BZ (2023). Design of one-for-all near-infrared aggregation-induced emission nanoaggregates for boosting theranostic efficacy. ACS Nano.

[68] He Z, Zhou H, Zhang Y, Du X, Liu S, Ji J, Yang X, Zhai G (2022). Oxygen-boosted biomimetic nanoplatform for synergetic phototherapy/ferroptosis activation and reversal of immune-suppressed tumor microenvironment. Biomaterials.

[69] Chen J, Gong M, Fan Y, Feng J, Han L, Xin HL, Cao M, Zhang Q, Zhang D, Lei D, Yin Y (2022). Collective plasmon coupling in gold nanoparticle clusters for highly efficient photothermal therapy. ACS Nano.

[70] Yu J, Wang L, Ling Y, Xiao X, Gong J, Jin H, Xu J, Chen P, Xie X, Zhang L (2023). Peptide-modified bioresponsive chondroitin sulfate micelles for targeted doxorubicin delivery in triple-negative breast cancer. Colloids Surf B..

[71] Zhang C, Ren J, He J, Ding Y, Huo D, Hu Y (2018). Long-term monitoring of tumor-related autophagy in vivo by Fe_3_O_4_-NO· nanoparticles. Biomaterials.

[72] Gu H, Liu W, Zhen S, Long S, Sun W, Cao J, Zhao X, Du J, Fan J, Peng X (2021). “Internal and external combined” nonradiative decay-based nanoagents for photoacoustic image-guided highly efficient photothermal therapy. ACS Appl Mater Interfaces.

[73] Wen K, Tan H, Peng Q, Chen H, Ma H, Wang L, Peng A, Shi Q, Cai X, Huang H (2022). Achieving efficient NIR-II type-I photosensitizers for photodynamic/photothermal therapy upon regulating chalcogen elements. Adv Mater.

[74] Cai Y, Wei Z, Song C, Tang C, Huang X, Hu Q, Dong X, Han W (2019). Novel acceptor-donor-acceptor structured small molecule-based nanoparticles for highly efficient photothermal therapy. Chem Commun.

[75] Zhang C, Xia D, Liu J, Huo D, Jiang X, Hu Y (2020). Bypassing the Immunosuppression of myeloid-derived suppressor cells by reversing tumor hypoxia using a platelet-inspired platform. Adv Funct Mater.

