# Supplementary material for: A novel polymer enabled by polymerized small molecule strategy for tumor photothermal and photodynamic therapy
Source: J Nanobiotechnology. 2023 Dec 20;21:497. doi: 10.1186/s12951-023-02272-9 (PMC10734082; doi:10.1186/s12951-023-02272-9)
Supplement: Supplementary file 1 — Additional file 1: Figure S1. 1H NMR spectrum of PYT in CDCl3. Figure S2. GPC spectra of PYT in TCB, estimated by high temperature gel permeation chromatography with 150 °C. Figure S3. Diameters of PYT NPs after storage in a water, b acidic PBS, c PBS and d serum for different times. Figure S4. The temperature changes of PYT NPs (50 μg mL−1) were recorded under 808 nm laser irradiation with different laser powers. Figure S5. UV–Vis-NIR spectra of PYT NPs during continuous 808 nm laser irradiation (1.0 W cm−2) for 0, 3,6, 9 min, respectively. Figure S6. Time-related ROS generation of ICG mixed with DPBF under laser power density (808 nm laser, 1.0 W cm−2). Figure S7. The photographs of PYT NPs (right) and ICG (left) before and after five repeated ON/OFF cycles irradiation with 808 nm lasers (1.0 W cm−2), respectively. Figure S8. Cellular uptake efficiency of FITC-labeled PYT NPs in 4T1 cells. Scale bar: 100 μm. Figure S9. Cell viability of a 4T1, b B16F10 and c HeLa after treatment with various concentrations of PYT NPs under dark condition for 48 h. “ns” means “no significance”, one-way ANOVA. Figure S10. Cell viability of a H9c2, b NIH 3T3 and c HEK 293 T after treatment with various concentrations of PYT NPs under dark condition for 48 h. n = 4, “ns” means “no significance”, one-way ANOVA. Figure S11. Cell viability of a 4T1, b HeLa and c B16F10 after treatment with different laser power values (808 nm). n = 4, “ns” means “no significance”, P-value: ****P < 0.0001, one-way ANOVA. Figure S12. Live/dead staining of 4T1 cancer cells after different treatments. Scale bar: 100 μm. Figure S13. Live/dead staining of Hela cancer cells after different treatments. Scale bar: 100 μm. Figure S14. Live/dead staining of B16F10 cancer cells after different treatments. Scale bar: 100 μm. Figure S15. In vitro wound scratch assay of 4T1 cells with different treatments. Scale bar: 100 μm. Figure S16. Expression of Bcl-2 and Bax in 4T1 cells incubated with PBS or PYT NPS (50 μg mL−1 [file 12951_2023_2272_MOESM1_ESM.docx]

**Additional file**

**A Novel Polymer Enabled by Polymerized Small Molecule Strategy for Tumor Photothermal and Photodynamic Therapy**

Xin Xie *^a,b^*, Ke Wang *^c^*, Jie Zeng *^a,b^*, Miao-Yan Xu *^a,b^*, Xin-Hui Qu *^d^*, Zheng-Bin Xiang *^d^*, Fang-Fang Tou *^e^*, Shaorong Huang *^a,b^* *, and Xiao-Jian Han *^b,d^* *

*^a^* Department of Pharmacology, School of Pharmaceutical Science, Nanchang University, Nanchang 330006, Jiangxi, People’s Republic of China.

*^b^* Institute of Geriatrics, Jiangxi Provincial People's Hospital & The First Affiliated Hospital of Nanchang Medical College, Nanchang 330006, Jiangxi, People’s Republic of China.

*^c^* Department of Clinical Laboratory, Jiangxi Provincial Children’s Hospital, Nanchang 330006, Jiangxi, People’s Republic of China.

*^d^* The Second Department of Neurology, Jiangxi Provincial People’s Hospital, the First Affiliated Hospital of Nanchang Medical College, Nanchang 330006, Jiangxi, People’s Republic of China.

*^e^* Department of Oncology, Jiangxi Provincial People’s Hospital, the First Affiliated Hospital of Nanchang Medical College, Nanchang 330006, Jiangxi, People’s Republic of China.

*Corresponding authors:

E-mail: hanxiaojian@hotmail.com (Xiao-Jian Han)

huangshaorong@ncmc.edu.cn (Shaorong Huang)

### Experimental Section

#### Materials and Instrumentation

All the starting materials were obtained from Sigma Aldrich, J&K Chemical Company (Shanghai). Commercially available reagents were used without further purification, unless noted otherwise. All chemicals were reagent grade or better. Y5 and 2,5-bis(triMethylstannyl)thiophene (T) were purchased from Derthon Optoelectronics Materials Science Technology Co LTD.

The ^1^H NMR spectra is recorded in deuterated solvents on a Bruker ADVANCE 400 NMR Spectrometer. ^1^H NMR chemical shifts is reported in ppm downfield from tetramethylsilane (TMS) reference using the residual protonated solvent as an internal standard. UV-vis absorption spectra are recorded on a Perkin Elmer Lambda 750 spectrophotometer. Transmission electron microscopy (TEM) was conducted on a JEM-ARM 200F Atomic Resolution Analytical Microscope operating at an accelerating voltage of 200 kV. Dynamic light scattering (DLS) was carried out with a NANO ZS (Malvern Panalytical Co., UK) at a fixed angle of 90°. The cellular fluorescence images were carried out using a confocal laser scanning microscope (CLSM, ZEISS-LSM900). In this work, cell counting kit-8 (CCK-8) was purchased from GLPBIO (United States) and the absorbance of each sample was detected at 450 nm with a microplate reader (Multiskan SkyHigh, Thermo Scientific™). The cell apoptosis was analyzed with a BD FACSAria SORP fluorescence activated cell sorting (FACS). Phototherapeutic experiments were carried out by an 808 nm infrared semiconductor laser (Changchun radium photoelectric technology). Temperature variation and photothermal images were taken by an E54 camera (FLIR System). Calcein acetoxymethylester and propidium iodide were purchased from Beijing Solarbio Science & Technology. The mitochondrial membrane potential assay kit with JC-1 were purchased from MedChemExpress. DSPE-PEG_2000_ and DSPE-PEG_2000_-FITC was obtained from Chongqing Yusi Pharmaceutical Technology Ltd. Fetal bovine serum were purchased from Viva cell biosciences. Ki67 antibody (#12202) was purchased from Cell signaling technology. Annexin-V PE/7-AAD apoptosis detection kit were purchased from BD Biosciences. MitoSOX (M36008) was purchased from Thermo Fisher Scientific Inc.

#### Synthesis of PYT NPs

PYT (5 mg) was dissolved in 2 mL THF, and DSPE-PEG_2000_ (5 mg) was completely dissolved in water (5 mL) to obtain DSPE-PEG_2000_ solution. Then the PYT solution was added into DSPE-PEG_2000_ solution under sonication (time: 10 min, power: 220 W). After 10 min sonication, the organic solvent was removed under N_2_ flow. The crude PYT NPs were further filtered through a membrane filter (diameter = 220 nm) and stored in dark at 4 °C for further usage.

#### Infrared thermal imaging of PYT NPs

The PYT NPs with 50 μg mL^-1^ in plastic tubes were irradiated by a 808 nm laser at a power density of 1.0 W cm^-2^ for 5 min. The real-time temperatures and infrared images were recorded using an infrared camera (Changchun, China) and analyzed using IR Flash thermal imaging analysis software (Infrared Cameras Inc.).

#### Photothermal conversion test and photostability of PYT NPs

The PYT NPs (0, 10, 30 and 50 μg mL^-1^) were exposed to an NIR laser (808 nm, 0.1, 0.4, 0.7 and 1.0 W cm^-2^, 5 min, laser on). Subsequently, the NIR laser was turned off, and the solution was naturally cooled to room temperature (laser off). The laser on and laser off cycles were repeated six times, and the change in temperature was monitored as described above, and then calculated of the photothermal conversion efficiency (η), PYT NPs (50 μg mL^-1^) were exposed to 808 nm laser (1.0 W cm^-2^). After heating, the solution was cooled down to room temperature. The *η* was calculated based on the reported method [1]:

$$\eta=\frac{hS \left( T_{max}-T_{surr} \right)-Q_{dis}}{I(1-{10}^{-A_{\lambda}})}$$

Where, *h* and *S* are heat transfer coefficient and surface area of the container, respectively; Q_dis_ is the heat dissipation from the laser mediated by the solvent and container; *I* and *A* are the laser power and absorption at 808 nm.

$$hS=\frac{mC_{water}}{\tau_{s}}$$

Where *m* is the mass of the PYT NPs solution, *C* is the specific heat capacity of the solution (*C_water_* = 4.2 J/g∙ºC), and τ_s_ is the associated time constant.

𝑡*=−*$\tau_{s}ln (\theta)$

Where *θ* is a dimensionless parameter, deduced from the temperature.

$$\theta=\frac{T-T_{surr}}{T_{max}-T_{surr}}$$

*T_max_* and *T_surr_* denote the maximum steady state temperature and the environmental temperature, respectively.

#### Reactive Oxygen Species (ROS)

PYT NPs (100 μL, 50 μg·mL^-1^) and Indocyanine green (ICG) aqueous solution (100 μL, 50 μg·mL^-1^) in quartz cuvettes were mixed with Detection 1,3-Diphenylisobenzofuran (DPBF) in ethanol (30.0 μL, 10 mM) respectively, which was then irradiated by lasers (808 nm, 1.0 W cm^-2^) for a period of 35 s. The absorbance at 415 nm of the solution was recorded every 7 s during the process.

#### Cell culture

The mouse cancer cell line 4T1, B16F10 and HeLa were obtained from the American Type Culture Collection (ATCC). The cells were cultured in complete RPMI 1640 medium (containing 10% FBS) at 37 °C in a 5% CO_2_ atmosphere.

#### Cellular uptake of fluorescent Micelles

To study the intracellular internalization behavior of PYT NPs by common confocal laser scanning microscopy, FITC-labeled PYT NPs (To simplify the following description, we abbreviate FITC-labeled PYT NPs as F-PYT NPS) were prepared through co-assembling DSPE-PEG_2000_ and DSPE-PEG_2000_-FITC (mole ratio is 9:1) with PYT via a nanoprecipitation method. 4T1 cells seeded at a suitable density in glass bottom dish were cultured for 24 h. Then, the cells were incubated with fresh 1640 medium containing F-PYT NPs (50 μg mL^-1^) at different times and washed with PBS. Afterwards, the samples and imaged by fluorescent microscope to observe the subcellular localization of PYT NPs. The excitation wavelengths are 488 nm for F-PYT NPs. The emission filters are 500~550 nm for F-PYT NPs.

#### Scratch plate assay

Scratch experiment was used to detect the effect of PYT NPs on 4T1 cell migration. The experiment was divided into four groups: PBS group, NIR group, PYT NPs group and PYT NPs + NIR group. Each group of cells in a logarithmic growth phase was inoculated in a 6-well plate at a rate of 10^6^ cells/well for culture. The monolayer cell surface was scratched vertically with a 10 μL gun head and then washed twice with PBS solution. After 0 h and 24 h, the cell migration around the scratched area was observed under the inverted microscope, and photos were taken.

#### Intracellular ROS generation

4T1 cells seeded in a six-well plate were cultured for 24 h. Then, the cells were treated with PYT NPs (50 μg mL^-1^) for 12 h. Afterwards, the cells were incubated with 1 mL fresh serum- free medium containing DCFH-DA (10 μM) at 37 °C for 30 min [2]. After washing, the cells were irradiated by 808 nm laser (1.0 W cm^-2^) for 5 min and then incubated at 37 °C for another 30 min. The cells were imaged by a Nikon Ti2 inverted fluorescence microscope.

#### Mito-SOX

The experiment was divided into four groups: PBS group, NIR group, PYT NPs group and PYT NPs + NIR group. Each group of cells in a logarithmic growth phase was inoculated in a 6-well plate at a rate of 10^5^ cells/well for culture. Take out the 4T1 tumor cells from the Cell Culture Incubator, suck out the RMPI 1640 medium with negative pressure aspirator, and rinse three times with PBS for 2 min. Add 2 μm Mito-SOX solution (dissolved in RMPI1640 medium) to each well, and the cells were left to incubate for 15 min at 37 °C in the dark. Each group was rinsed with PBS solution for 3 times prior to visualization. Each group was observed and photographed by inverted fluorescence microscope.

#### Measurement of MMP with the JC-1 assay

Mitochondrial membrane potentials (MMPs) were determined by the retention of the JC-1 assays. 4T1 cells were seeded on 35 mm glass dishes at a density of 2 × 10^5^ cells per well. The cells were treated with various nanocomposites for 12 h and then exposed to 808 nm laser irradiation for 5 min at a power density of 1.0 W cm^-2^. After another incubation for 12 h, the JC-1 relevant probes were added to the medium and incubated for 25 min. Then the cells were washed with PBS at 4 °C three times. Finally, the MMPs of the 4T1 cells were captured by a Nikon Ti2 inverted fluorescence microscope.

#### Live-dead cell staining

4T1 cells were seeded in a six-well plate to adhere 24 h. Then, the cells were administrated with different treatments: PBS, 808 nm laser for 5 min irradiation, PYT NPs with 12 h incubation and 808 nm laser for 5 min irradiation of PYT NPs incubated with 12 h. The concentration of PYT NPs was 50 μg mL^-1^ and the laser power was 1.0 W cm^-2^. After that, the cells were incubated at 37 °C for another 30 min, following by staining with PI (2 μM) and Calcein-AM (9 μM) for 15 min [3]. Subsequently, the cells were gently washed and imaged by inverted epifluorescence microscope.

#### In *vitro* photo cytotoxicity of PYT NPs

4T1, B16f10 and HeLa cells were seeded in 96-well plate (3 × 10^3^ cells per well) at an atmosphere of 37 °C and 5% CO_2_ for 12 h. Then the medium was replaced with a fresh PYT NPs at different concentrations and the cells were incubated for another 12 h. The cells were washed with PBS for three times, added with fresh medium, and then exposed to the NIR laser (808 nm, 1.0 W cm^-2^) for 5 min. After that, the cells were incubated for additional 24 h and their viability was analyzed using CCK-8 assay.

#### Cell apoptosis detection

4T1 cells (10^6^ cells per well) were seeded and cultured in 6-well plates for 24 h. Then, PYT NPs (50 μg mL^-1^) were added into the culture media. After 12 h-incubation, the cells were collected and irradiated by 808 nm laser (1.0 W cm^-2^) for 5 min. The cells treated with NPs without laser irradiation or PBS with/without laser irradiation were taken as the controls. Afterwards, the cells were incubated at 4 °C for another 0.5 h. Subsequently, the cells were washed by PBS and collected through centrifugation at 1000 rpm for 5 min (4 °C). Then, the samples were stained with an Annexin V-FITC/7-AAD Kit based on manufacturer’s instructions and examined by FACS.

#### The expression levels of apoptosis-related proteins

Western blotting was performed to analyze the expression of apoptosis-related proteins. 4T1 cells were cultured in a 96-well plate at 3 × 10^3^ cells/well for 24 h and then treated with PYT NPs (50 μg mL^-1^) for 12 h. Afterwards, the cells were irradiated by 808 nm laser (1.0 W cm^-2^) for 5 min. Cells was harvested after irradiated 24 h, and the mixture was centrifuged (12000 rpm for 10 min) to obtain the supernatant as the cell lysate. Bradford protein analysis (Bio-Rad Laboratories) was used to quantify the concentration of the extracted proteins. The cell lysate was separated using 12% sodium dodecyl sulfate polyacrylamide gel electrophoresis, then transferred to nitrocellulose membranes (Hercules,CA, USA). After 1 h blocking of the membranes in 5% skim milk-TBST (20 mM Tris HCl, pH 7.5, 150 mM NaCl, 0.1% Tween-20), the target primary antibody Bcl-2 (1:500), Bax (1:1000) and β-actin (1:100000) were added for an overnight (4 °C) reaction. Next, rabbit IgG or mouse IgG (1:5000) was added for a 1 h reaction (21~25 °C). Afterwards, ECL detection reagents were used for blotting, while density was measured using Image J Launcher (provided by NCBI).

#### In Vitro Fluorescence Imaging

PYT NPs (0, 10, 20, 30, 40, 50 μg/mL, respectively) in PBS solutions (PH 7.4) were subjected to fluorescence imaging. All solutions were put in eppendorf tubes and fluorescence image is by a fluorescence imaging system (PerkinElmer IVIS Lumina) with excitation at 780 nm and emission at 845 nm.

#### Statements for the animal experiments

All the animals received tender care incompliance with the guidelines outlined in the Guide for the Care and Use of Laboratory Animals. The procedures (including the tumors of the mice were irradiated with 808 nm laser for 5 min at a power density of 1.0 W cm^-2^) were approved by the Ethics Committee of Jiangxi Provincial People’s Hospital (SYXK (gan) 2021-0001).

#### Animal tumor model

Female BALB/c mice (4~6 weeks old) were purchased from SPF Biotechnology Co., Ltd (Beijing, China). 4T1 cells (1 × 10^6^ /100 μL for each mouse) were injected into the right thigh of female BALB/c mice to establish a subcutaneous 4T1 tumor model [4]. The tumor-bearing mice used for subsequent experiments, until the tumor volume reached 80~100 mm^3^. Tumor volume was calculated as (tumor length) × (tumor width)^2^ × 0.5.

#### *In* *vivo* fluorescence imaging

The tumor-bearing mouse was anesthetized with isoflurane to remove fur. Next, the PYT NPs solution (200 μL, 1 mg·mL^-1^) was intravenously injected into the mouse. After injection, the mouse was imaged with the small animal imaging system (IVIS Lumina Series III, PerkinElmer) at designated time points.

#### Hemolysis assay

Fresh whole blood (1.0 mL) was extracted by enucleation of BALB/c mouse eyes, and the RBCs were obtained by centrifugation (3000 rpm) for 5 min at 4 °C. The RBCs were further washed by PBS and diluted by PBS as 10 mL. Then, 0.2 mL of the suspension was mixed with 0.8 mL of PYT NPs solutions in PBS (pH = 7.4) to obtain a different concentration of 0, 0.25, 0.5, 0.75 and 1.00 mg mL^-1^, respectively. Each sample was prepared by triplicate, and the suspensions were incubated at 37 °C for 2 h under mild shaking (50 rpm/min). Then, the samples were centrifuged at 3000 rpm for 5 min at 4 °C, and the absorbance at 541 nm from hemoglobin was monitored by a UV-Vis spectrometer. To achieve 0% and 100% hemolysis, 0.2 mL of diluted RBC suspension was treated with PBS (0.8 mL) or water (0.8 mL), respectively. The percent of hemolysis is calculated with the formula: Hemolysis rate (%)= (Abs-Abs0%)/(Abs100%-Abs0%) × 100%. Where Abs, Abs0% and Abs100% were the absorption of samples (with the absorption background of PYT NPs subtracted), the solution of 0% hemolysis and the solution of 100% hemolysis, respectively.

#### *In vivo* photothermal imaging studies

4T1 tumor-bearing mice were intravenously injected with PYT NPs as described above. To verify the photothermal effects of the PYT NPs, four groups (PBS, NIR, PYT NPs, and PYT NPs + NIR) were intravenously injected with PBS, or PYT NPs. Then, 0 h, 3 h, 6h, 9 h, 12 h and 24 h after injection, the tumors for each group were continuously irradiated by NIR laser-808 nm (1.0 W cm^-2^) for 5 min and a thermal imaging camera recorded the treatment process every 1 min. Finally, the infrared images of real-time temperatures were analyzed using IR Flash thermal imaging analysis software.

#### *In vivo* photothermal therapeutic efficacy of PYT NPs

When the tumors reached approximately 100 mm^3^, 4T1 tumor-bearing mice were randomly divided into four groups (n=4 per group) and were intravenously injected with PYT NPs (1 mg mL^-1^) or PBS. After 12 h of injection, the tumors were irradiated by an 808 nm laser at a power density of 1.0 W cm^-2^ for 5 min. The tumor sizes were measured by a vernier calipers every second day, and the mouse weights were recorded every second day. The detailed grouping is as follows: PBS group were intravenously injected with 200 μL PBS. PBS+NIR group were intravenously injected with 200 μL PBS and irradiated with 808 nm laser for 5 min at a power density of 1.0 W cm^-2^. PYT NPs group were intravenously injected with 1 mg mL^-1^ (200 μL) PYT NPs. PYT NPs + NIR group were intravenously injected with 1 mg mL^-1^ (200 μL) PYT NPs and irradiated with 808 nm laser for 5 min at a power density of 1.0 W cm^-2^.

#### Hematoxylin & Eosin (H&E) staining

At the end of photothermal therapy, the mice were sacrificed and major organs (liver, kidneys, spleen, lungs, heart) were excised, fixed in 4% paraformaldehyde for 12 h, and embedded in paraffin for histopathological examination. Cell state in tumor tissue was analyzed by hematoxylin-eosin (H&E) staining.

#### Immunohistochemical staining analysis

At the end of photothermal therapy, the mice were sacrificed and tumor tissue were excised, fixed in 4% paraformaldehyde for 12 h, and embedded in paraffin for histopathological examination. Cell proliferation in tumor tissue were also analyzed by immunofluorescence staining of the Ki67 antigen (#12202, diluted 400 times) at 4 °C overnight after deparaffinized and rehydrated. Secondary antibody (Biotin-conjugated Affinipure Goat Anti-Rabbit IgG, Proteintech, SA00004-2, diluted 500 times) were treated 1 h at room temperature. Finally, signals were developed with Hematoxylin and DAB (Dako, Agilent Technologies, USA).

#### Statistical analysis

All data were expressed as mean ± standard deviation (SD). All experiments were repeated at least 3 times. One-way analysis of variance (ANOVA) and student's *t* test were used to evaluate the statistical significance. *P* values < 0.05 were regarded statistically significant. Results were considered statistically significant when P < 0.05. The statistical analysis was performed with Origin 2021 software (OriginLab, Northhampton, MA) or GraphPad Prism (GraphPad Prism 8; GraphPad).


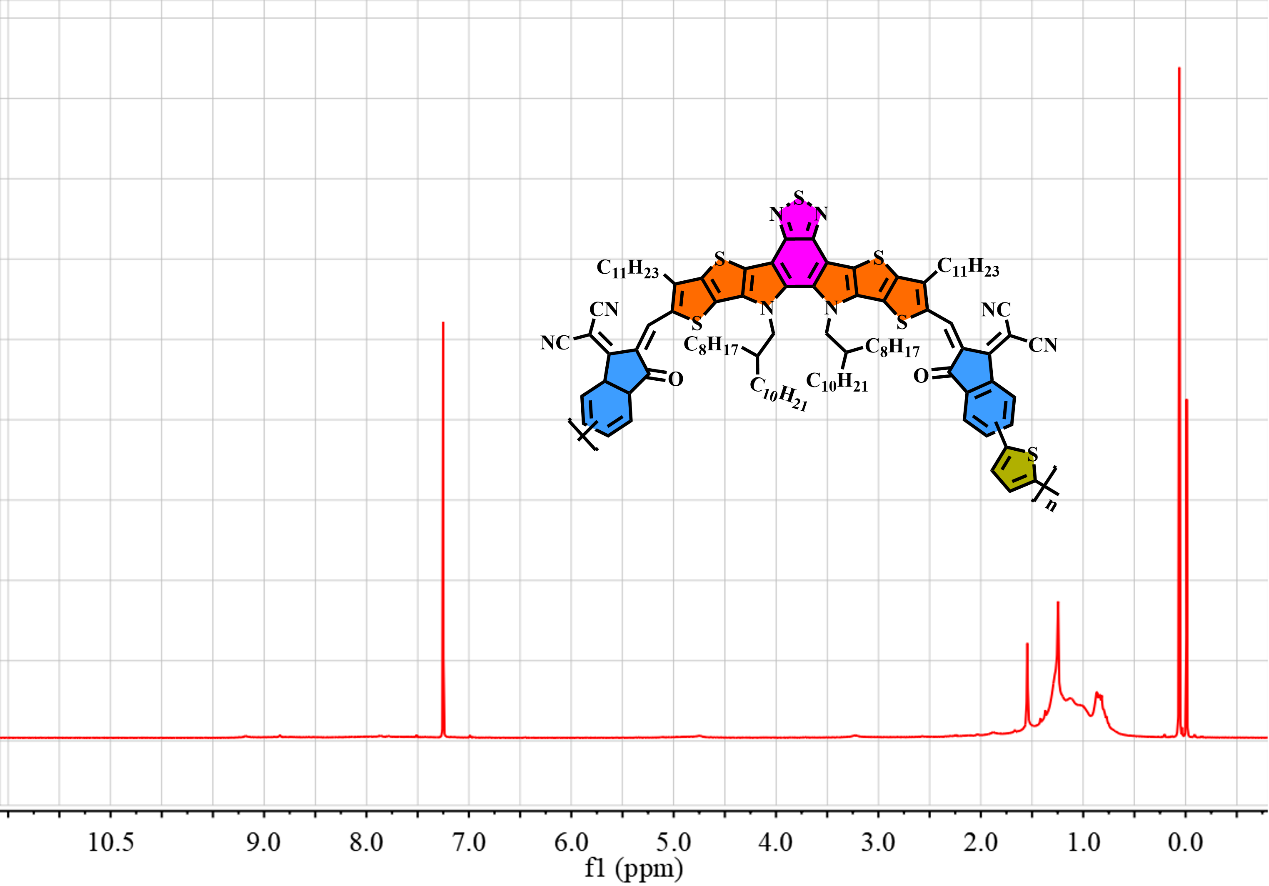
 **Figure S1** 1H NMR spectrum of PYT in CDCl3.


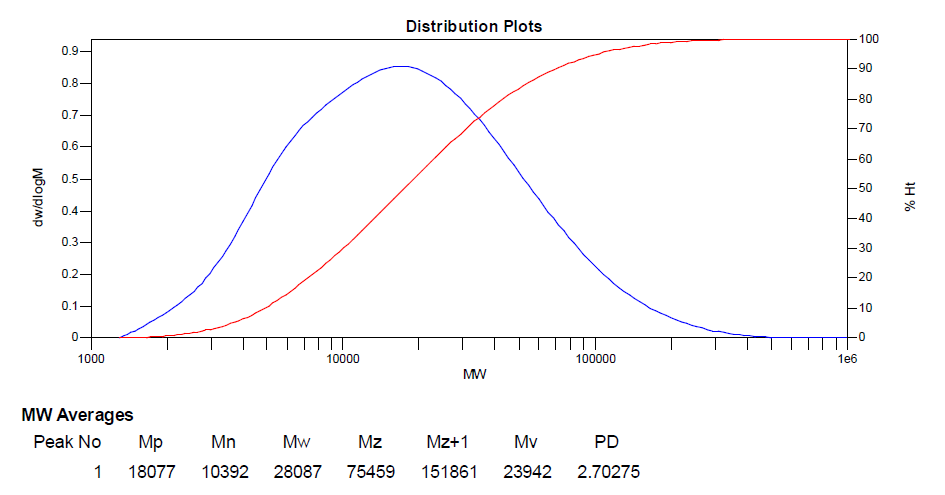
 **Figure S2** GPC spectra of PYT in TCB, estimated by high temperature gel permeation chromatography with 150 ºC.


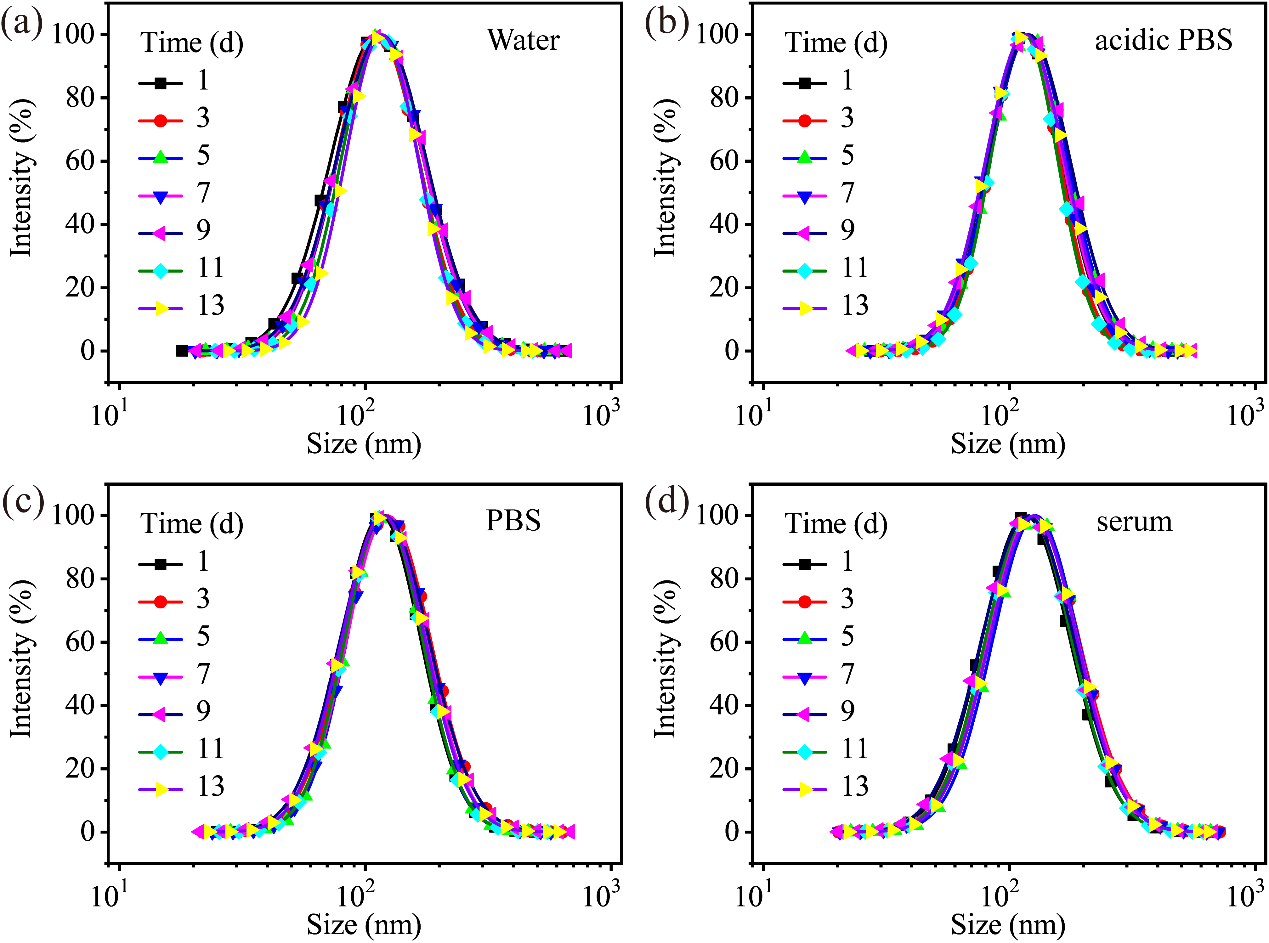

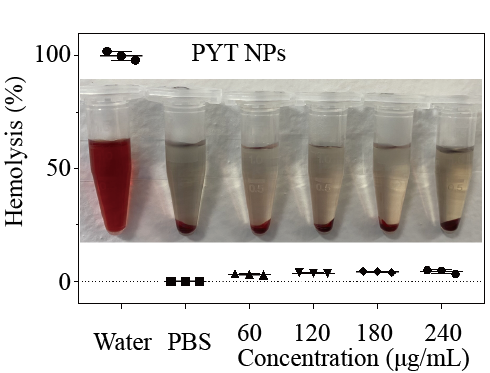


**Figure S3** Diameters of PYT NPs after storage in (a) water, (b) acidic PBS, (c) PBS and (d) serum for different times.


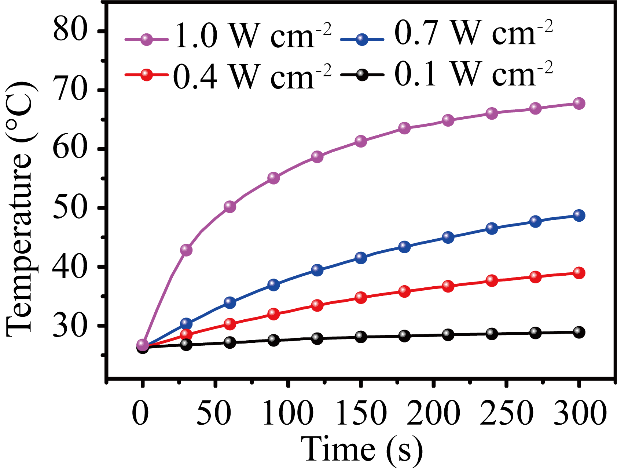


**Figure S4** The temperature changes of PYT NPs (50 μg mL^-1^) were recorded under 808 nm laser irradiation with different laser powers.


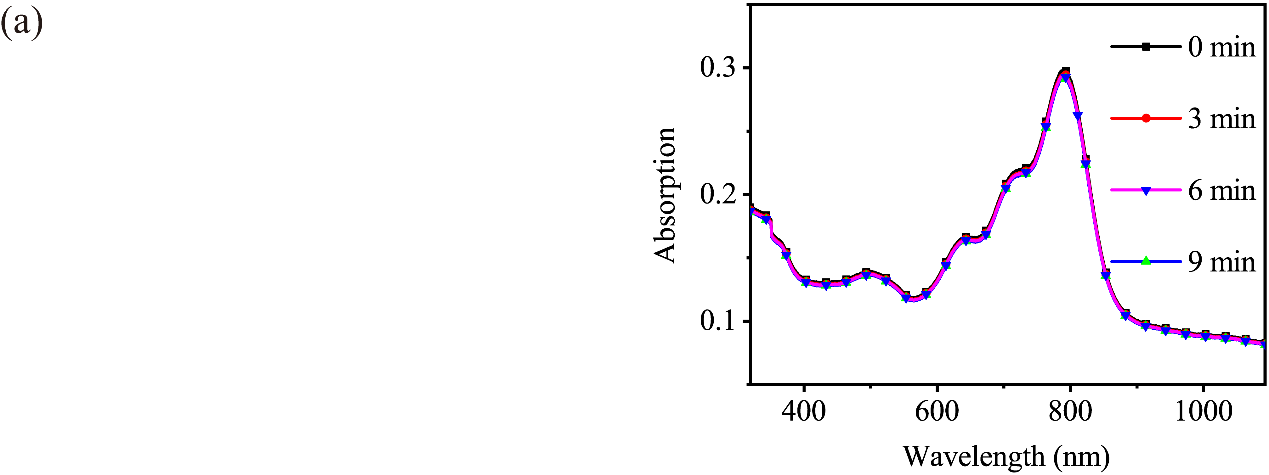


**Figure S5** UV-Vis-NIR spectra of PYT NPs during continuous 808 nm laser irradiation (1.0 W cm^-2^) for 0, 3,6, 9 min, respectively.


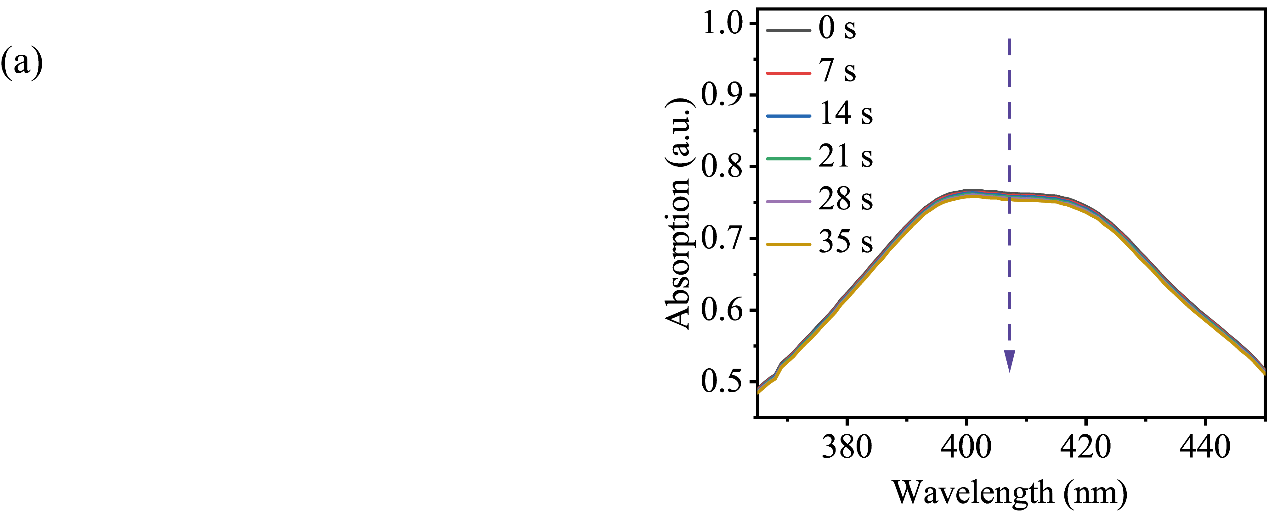


**Figure S6** Time-related ROS generation of ICG mixed with DPBF under laser power density (808 nm laser, 1.0 W cm^-2^).


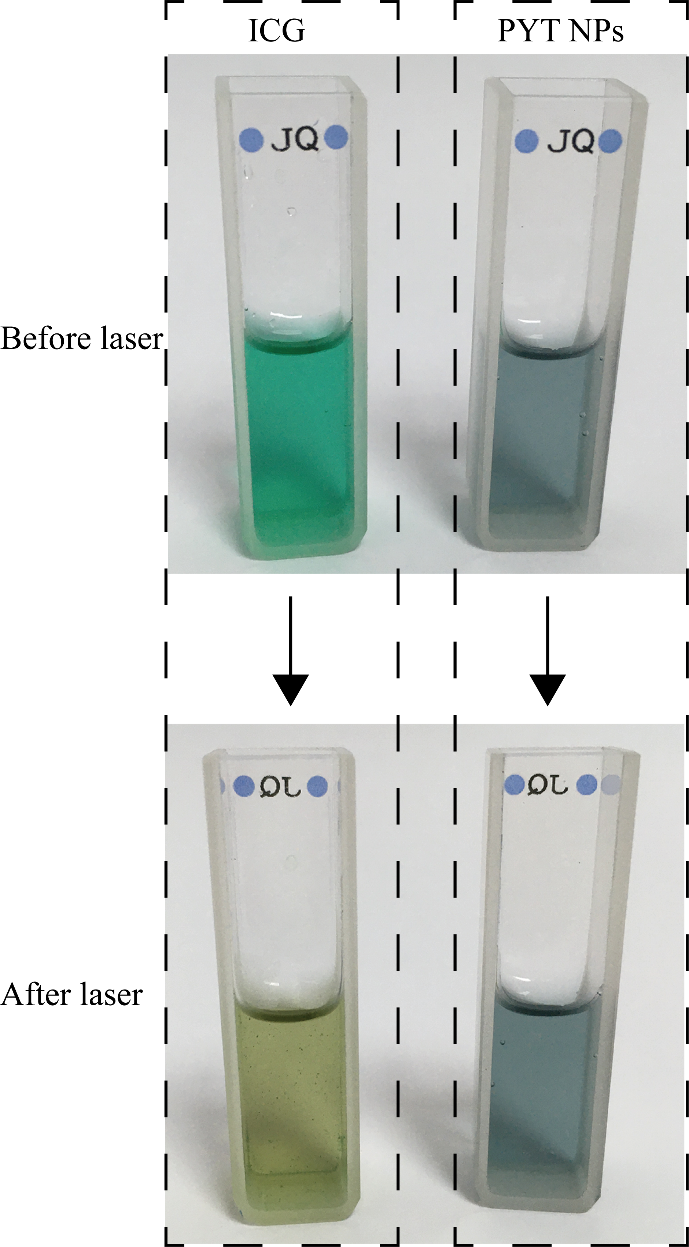


**Figure S7** The photographs of PYT NPs (right) and ICG (left) before and after five repeated ON/OFF cycles irradiation with 808 nm lasers (1.0 W cm^-2^), respectively.


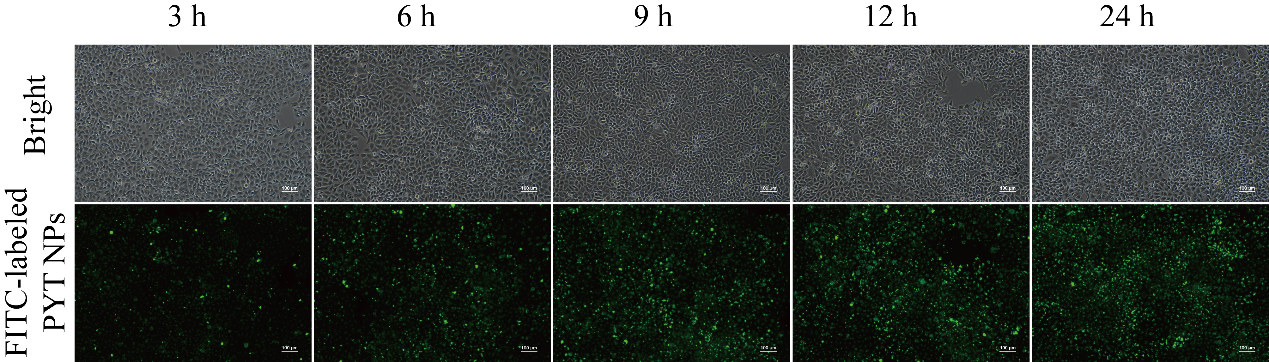


**Figure S8** Cellular uptake efficiency of FITC-labeled PYT NPs in 4T1 cells. Scale bar: 100 μm.


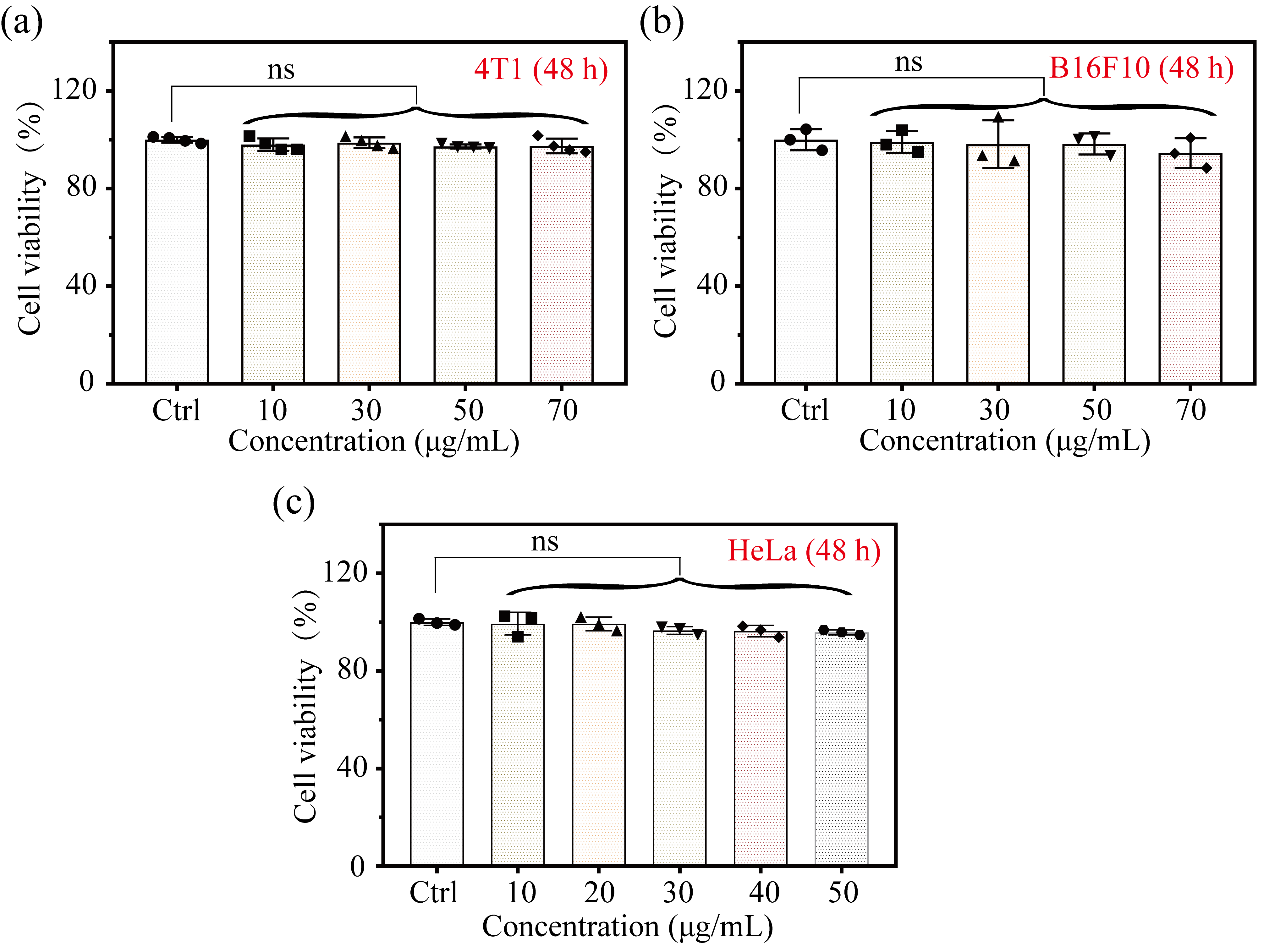


**Figure S9** Cell viability of (a) 4T1, (b) B16F10 and (c) HeLa after treatment with various concentrations of PYT NPs under dark condition for 48 h. “ns” means “no significance”, one-way ANOVA.


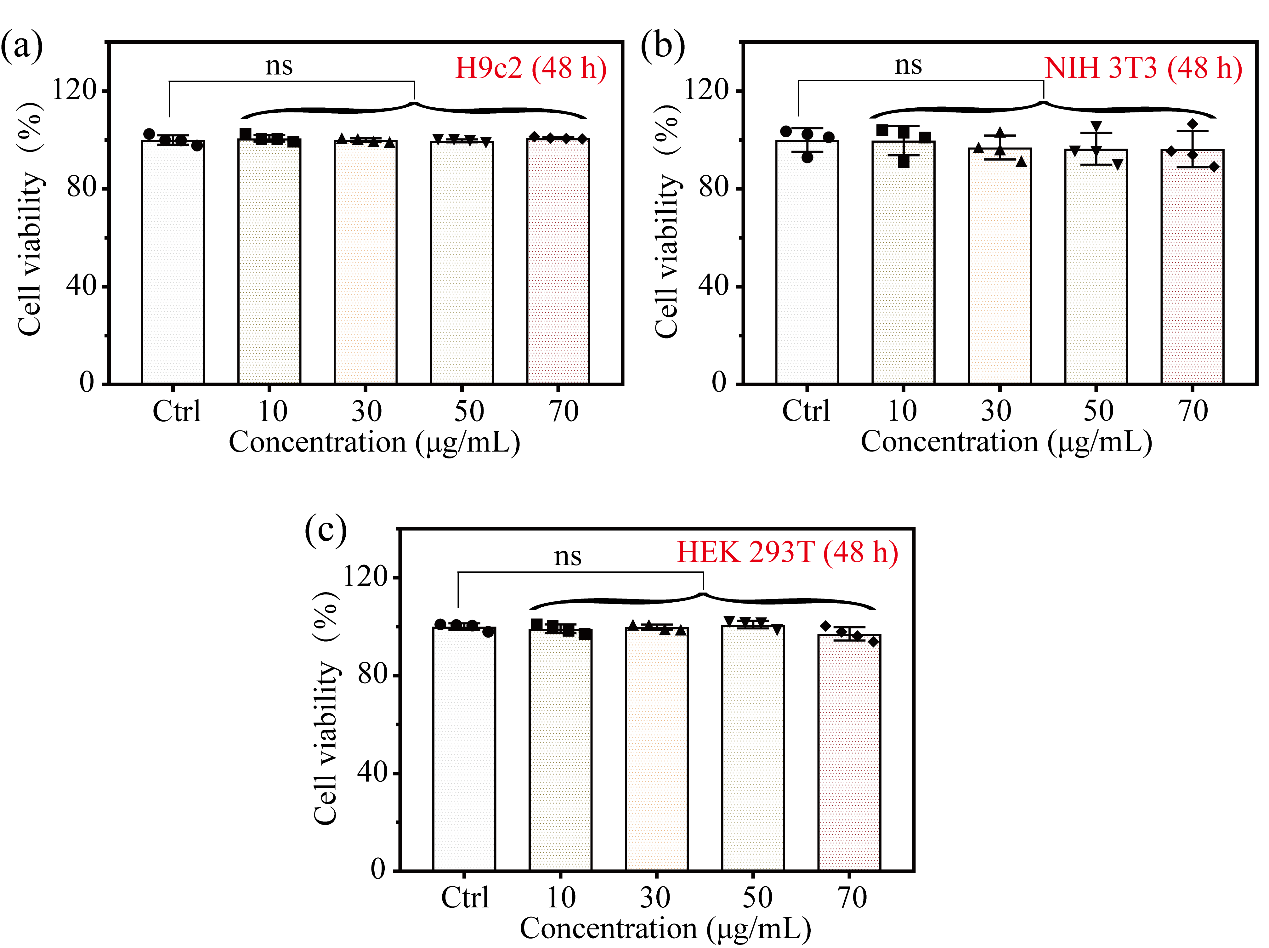


**Figure S10** Cell viability of (a) H9c2, (b) NIH 3T3 and (c) HEK 293T after treatment with various concentrations of PYT NPs under dark condition for 48 h. n = 4, “ns” means “no significance”, one-way ANOVA.


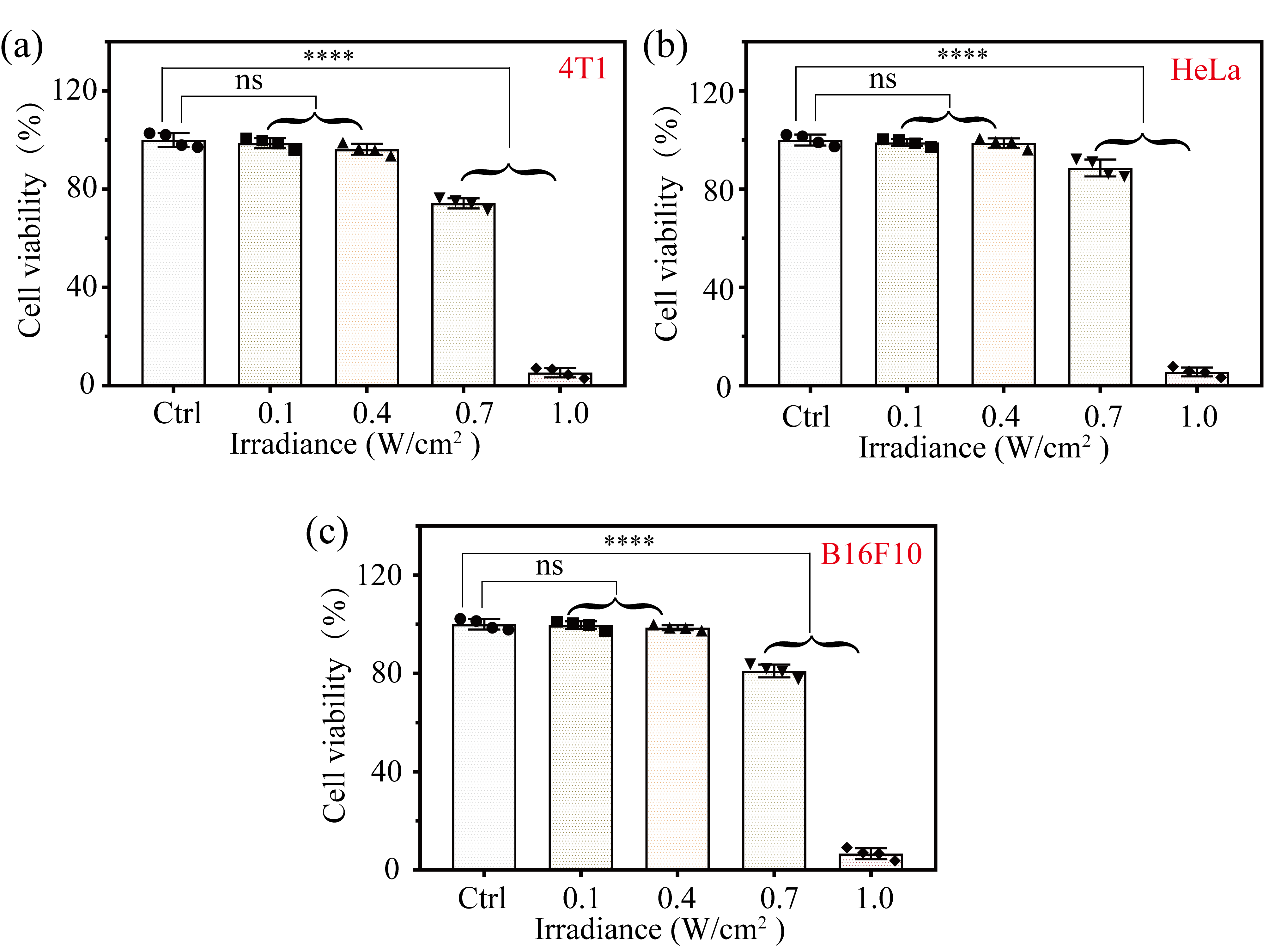


**Figure S11** Cell viability of (a) 4T1, (b) HeLa and (c) B16F10 after treatment with different laser power values (808 nm). n = 4, “ns” means “no significance”, *P*-value: *****P* < 0.0001, one-way ANOVA.


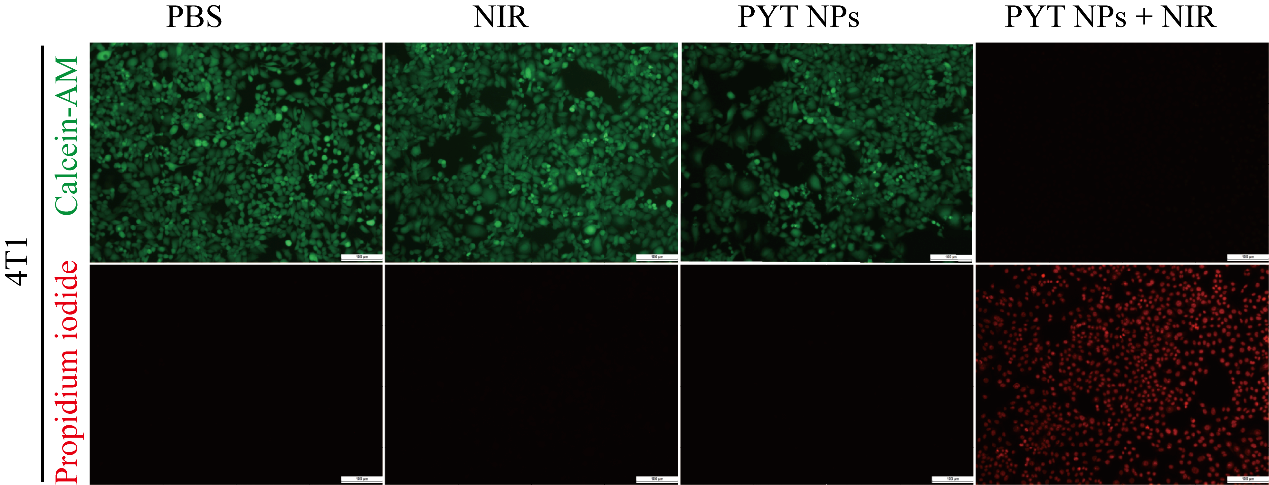


**Figure S12** Live/dead staining of 4T1 cancer cells after different treatments. Scale bar: 100 μm.


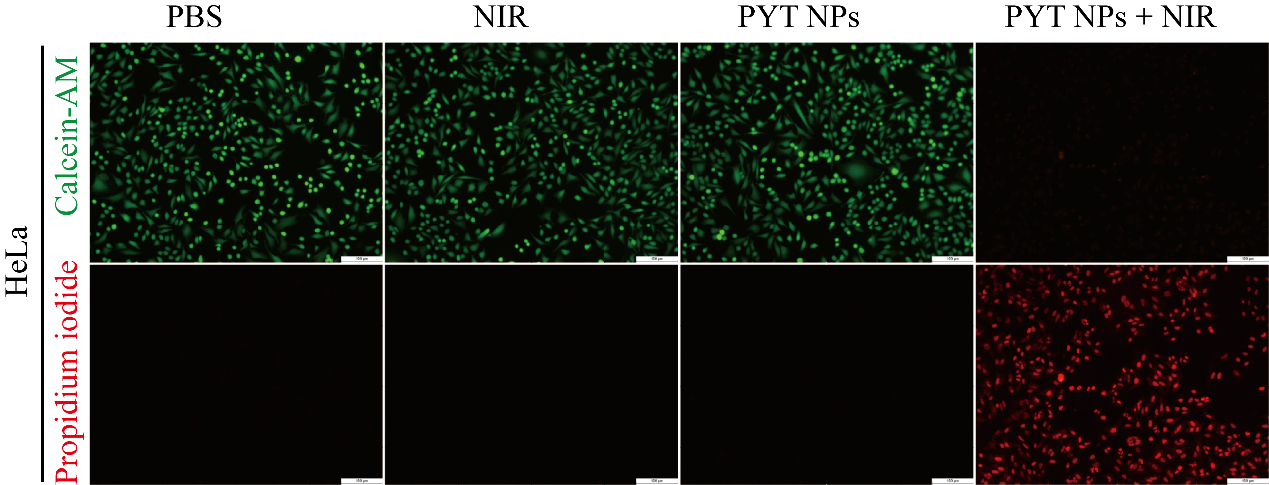


**Figure S13** Live/dead staining of Hela cancer cells after different treatments. Scale bar: 100 μm.


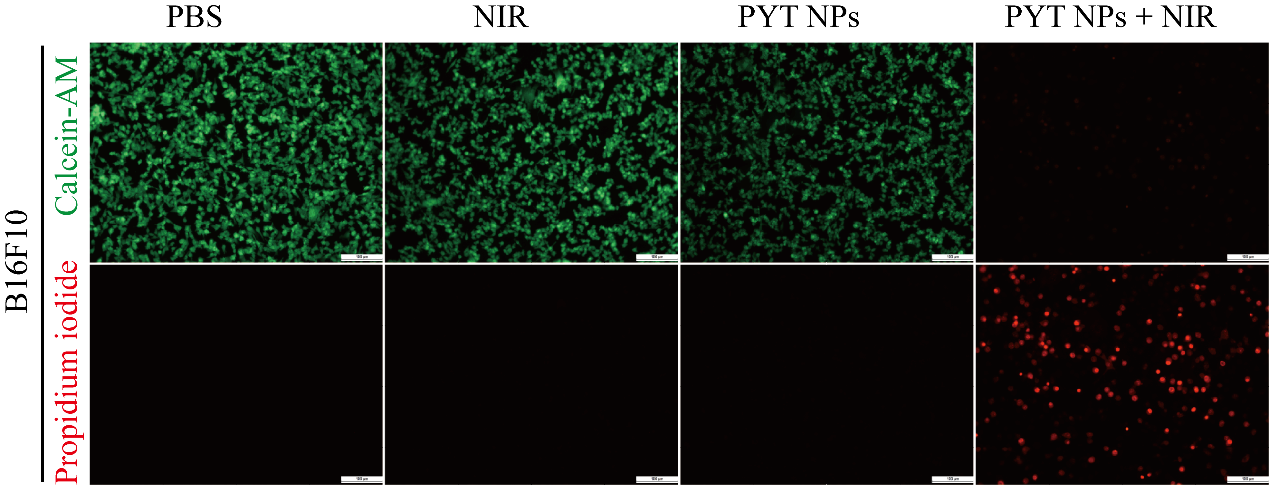


**Figure S14** Live/dead staining of B16F10 cancer cells after different treatments. Scale bar: 100 μm.


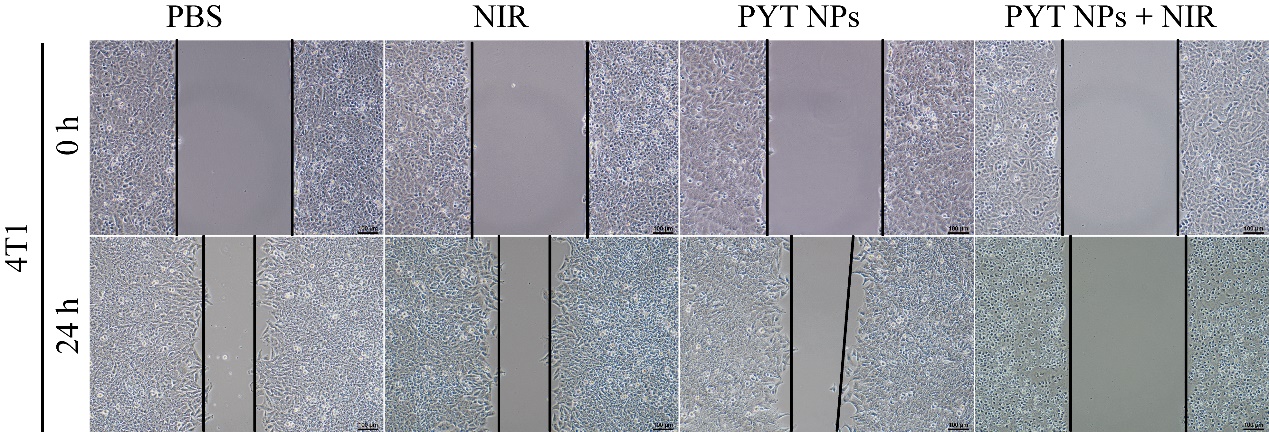


**Figure S15** *In vitro* wound scratch assay of 4T1 cells with different treatments. Scale bar: 100 μm.


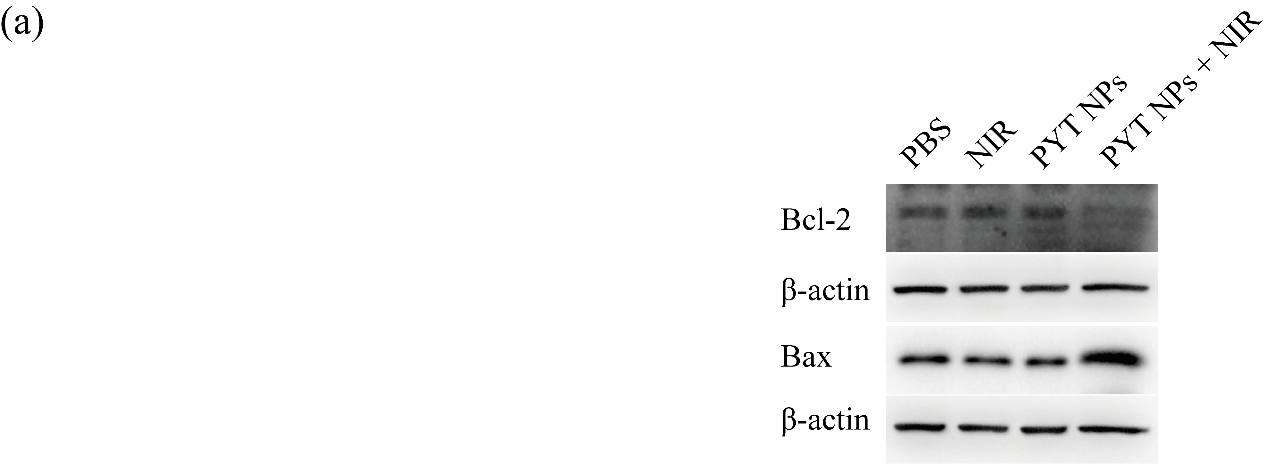


**Figure S16** Expression of Bcl-2 and Bax in 4T1 cells incubated with PBS or PYT NPS (50 μg mL^-1^ for PYT NPs) was detected by western blot analysis. β-actin was used as the internal control..


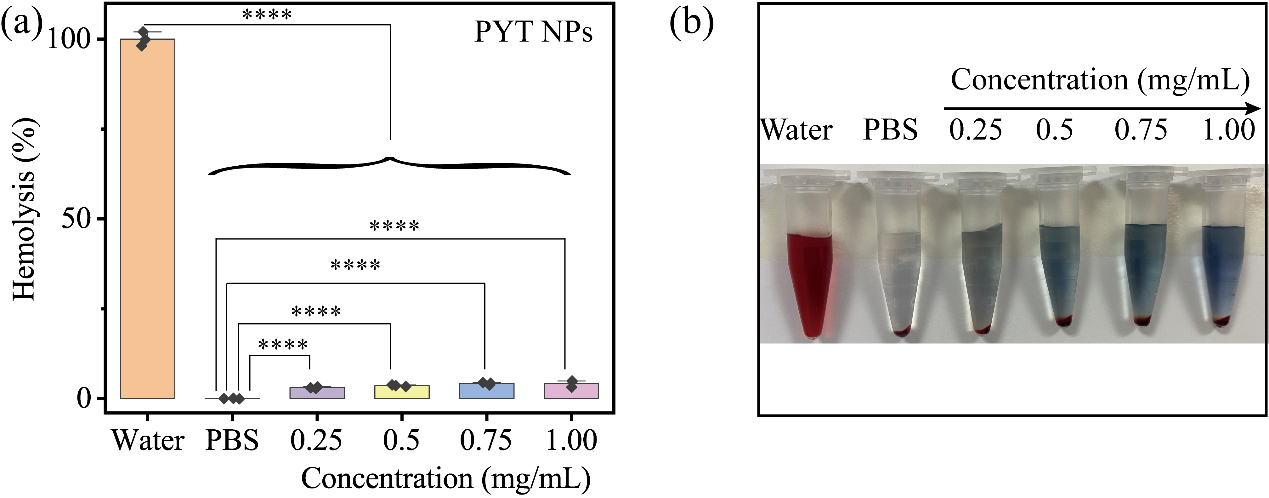


**Figure S17** (a) Hemolytic effect of PYT NPs at different concentrations on RBCs from BALB/c mice, water group was used as the positive control, and PBS as the negative control, respectively. (b) Representative photos of hemolysis experiment results. n = 3, “ns” means “no significance”, *P*-value: *****P* < 0.0001.


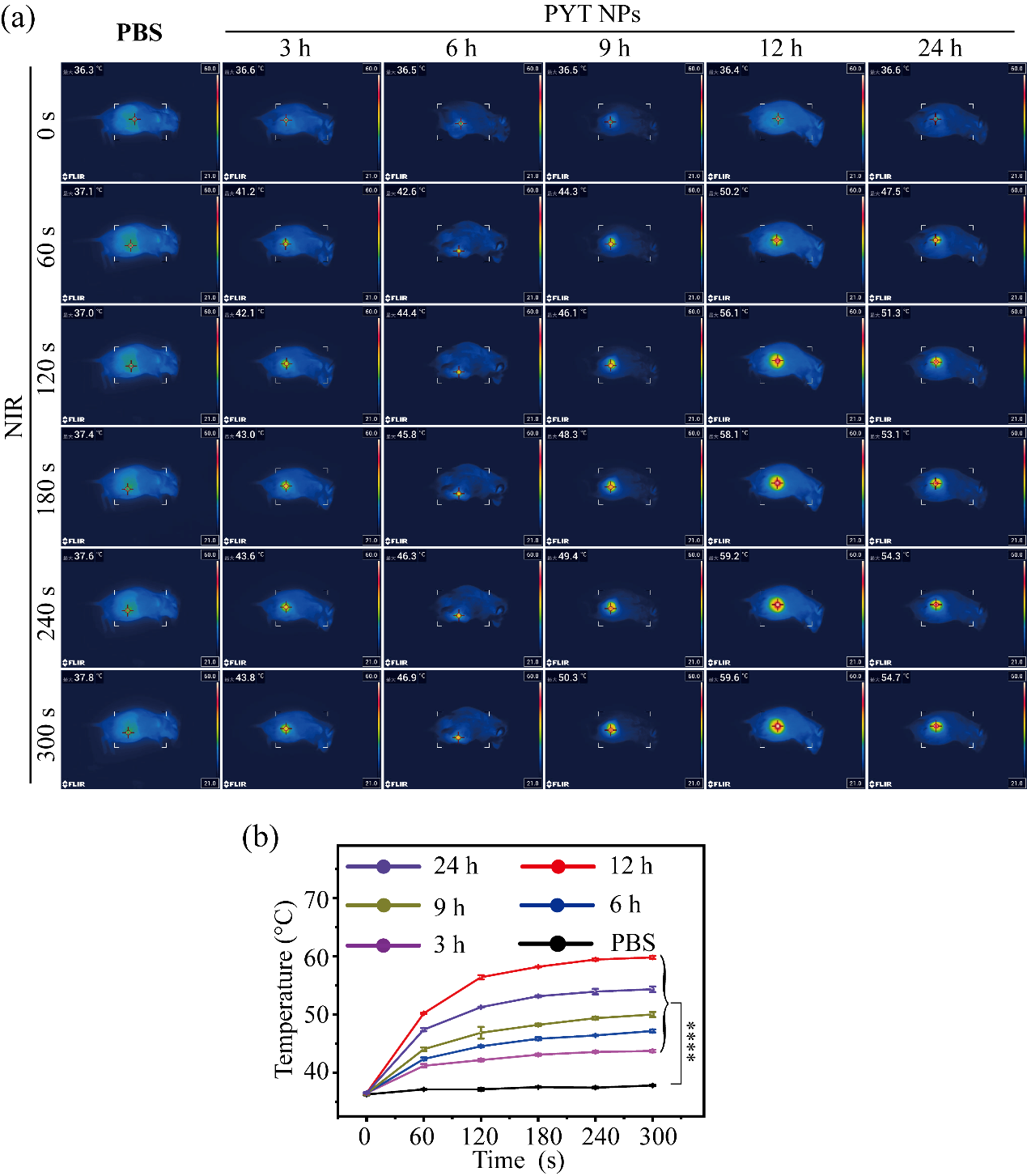


**Figure S18** (a) Infrared images were collected from 4T1 tumor-bearing BALB/c mice at different time intervals after 808 nm laser irradiation. (b) Taking temperature as the ordinate and time as the abscissa, the temperature curve of laser-heated mouse tumor site was drawn. n = 3, *****P* < 0.0001, one-way ANOVA.


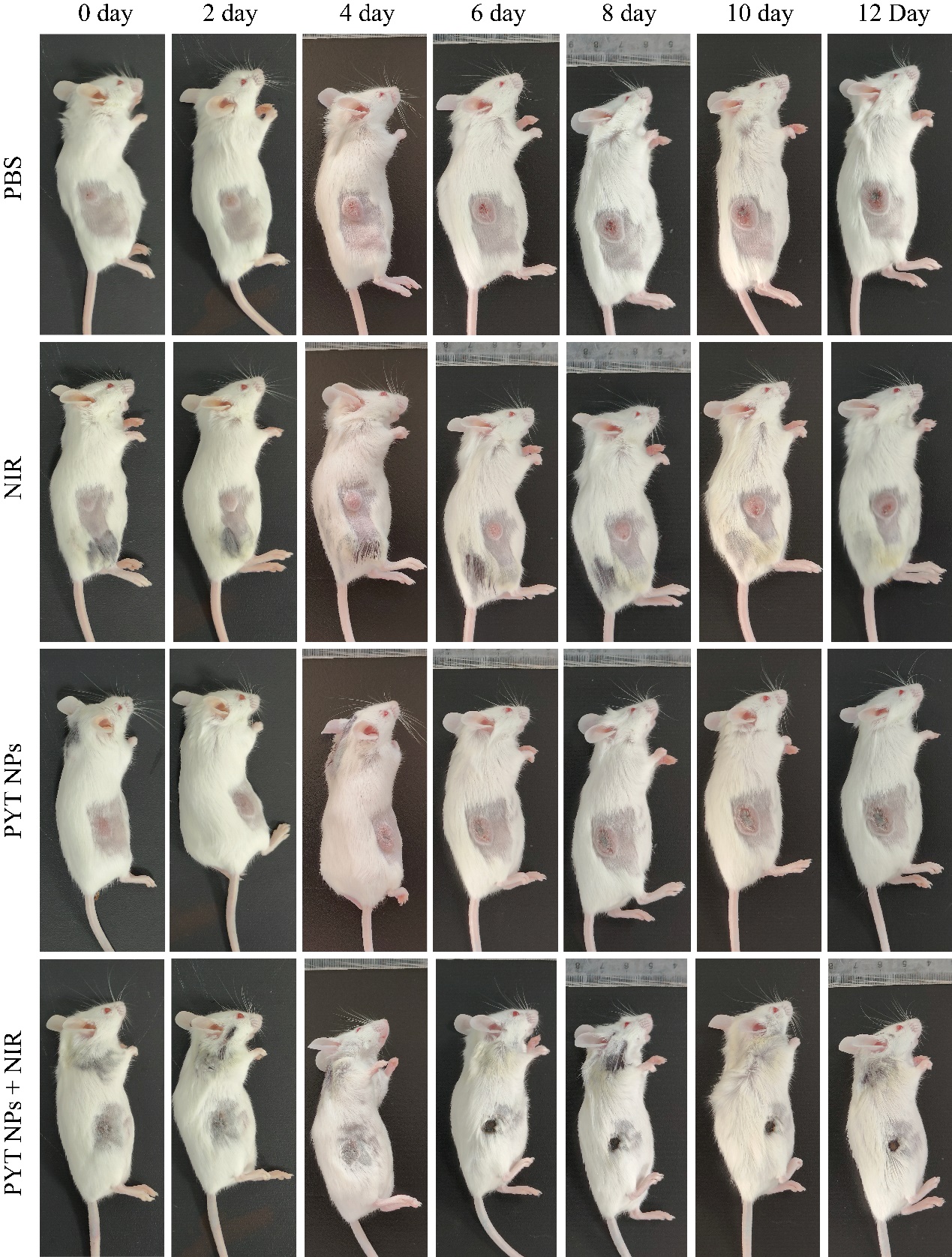


**Figure S19** Representative pictures of photothermal therapy for 4T1 tumor bearing mice after different treatments (PBS, NIR, PYT NPs and PYT NPs + NIR) in vivo.

**Table S1** Hepatic function indexes (ALB, ALT and AST) and renal function markers (UREA, CREA and UA) of mice at day 12 after treatment.

|  |  | Saline | PYT NPs + NIR | Reference range |
| --- | --- | --- | --- | --- |
| ALB | g/L | 27.4±1.9 | 28.4±1.0 | 21.22~39.15 |
| ALT | U/L | 43.6±5.4 | 51.3±16.9 | 10.06~96.47 |
| AST | U/L | 27.4±1.9 | 28.4±1.0 | 36.31~235.48 |
| UREA | mmol/L | 1.85±0.2 | 1.89±0.1 | 0.6~1.93 |
| CREA | μmol/L | 43.3±15.1 | 27.0±2.2 | 10.91~85.09 |
| UA | μmol/L | 78.5±30.9 | 103.3±42.0 | 44.42~224.77 |

**Table S2** Routine blood indexes of mice at day 12 after treatment.

|  | Saline | PYT NPs + NIR | Reference range |
| --- | --- | --- | --- |
| WBC (10^9^/L) | 4.8±0.5 | 5.1±1.1 | 0.8~6.8 |
| NEU (10^9^/L) | 0.8±0.1 | 1.1±0.3 | 0.1~1.8 |
| LYM (10^9^/L) | 3.7±0.4 | 3.9±1.0 | 0.7~5.7 |
| MON (10^9^/L) | 0.2±0.1 | 0.1±0.1 | 0.0~0.3 |
| RBC(10^12^/L) | 7.0±0.9 | 9.0±0.4 | 6.36~9.42 |
| HGB(g/L) | 128±10.7 | 120±6.8 | 110~143 |
| HCT(%) | 41.9±2.1 | 40.6±1.9 | 34.6~44.6 |
| MCV(fL) | 54.5±3.3 | 49.2±0.4 | 48.2~58.3 |
| MCH(pg) | 18.2±1.4 | 16.9±0.8 | 15.8~19 |
| PLT(10^9^/L) | 541.3±56.7 | 524±18.9 | 450~1590 |
| MPV(fL) | 5.5±0.4 | 5.4±0.3 | 3.8~6.0 |

References

1. Lin R, Liu J, Xu W, Liu Z, He X, Zheng C, Kang M, Li X, Zhang Z, Feng H-T, Lam JWY, Wang D, Chen M, Tang BZ. Type I photosensitization with strong hydroxyl radical generation in nir dye boosted by vigorous intramolecular motions for synergistic therapy. *Adv. Mater.* 2023; 35:2303212.

2. Zhang Z, Ding D, Liu J, Huang C, Li W, Lu K, Cheng N. Supramolecular Nanozyme System Based on Polydopamine and Polyoxometalate for Photothermal-Enhanced Multienzyme Cascade Catalytic Tumor Therapy. *ACS Appl. Mater. Interfaces* 2023; 15:38214-38229.

3. Wang M, Li Y, Wang M, Liu K, Hoover AR, Li M, Towner RA, Mukherjee P, Zhou F, Qu J, Chen WR. Synergistic interventional photothermal therapy and immunotherapy using an iron oxide nanoplatform for the treatment of pancreatic cancer. *Acta Biomater*. 2022; 138:453-462.

4. Ma G, Liu Z, Zhu C, Chen H, Kwok RTK, Zhang P, Tang BZ, Cai L, Gong P. H_2_ O_2_-responsive NIR-II AIE nanobomb for carbon monoxide boosting low-temperature photothermal therapy. *Angew. Chem. Int. Ed.* 2022; 61:e202207213.
